# Exploring community insights on antimicrobial resistance in Nepal: a formative qualitative study

**DOI:** 10.1186/s12913-023-10470-2

**Published:** 2024-01-11

**Authors:** Ayuska Parajuli, Lidis Garbovan, Basudev Bhattarai, Abriti Arjyal, Sushil Baral, Paul Cooke, Sophia Latham, Dani J. Barrington, Jessica Mitchell, Rebecca King

**Affiliations:** 1 HERD International, Bhaisepati, Lalitpur, Nepal; 2 Centre for World Cinema and Digital Cultures, University of Leeds, Woodhouse Lane, Leeds, LS2 9JT UK; 3 Institute of Infection, Veterinary and Ecological Sciences, University of Liverpool, Chester High Road, Neston, Liverpool, CH64 7TE UK; 4 School of Population and Global Health, University of Western Australia, 35 Stirling Highway, Crawley, Perth, Western 6009 Australia; 5 Nuffield Centre for International Health and Development, University of Leeds, Woodhouse Lane, Leeds, LS2 9JT UK

**Keywords:** Antimicrobial resistance, Community, Nepal, Qualitative study, Drivers of antimicrobial resistance

## Abstract

**Background:**

Antimicrobial resistance (AMR) is the process by which microbes evolve mechanisms to survive the medicines designed to destroy them i.e. antimicrobials (AMs). Despite being a natural process, AMR is being hastened by the abuse of AMs. In context of Nepal, there is limited information on drivers of AMR and barriers in addressing it from a community perspective. This study explores the local language and terminology used around AMs in the community, commonly used AMs and reasons for their usage, how these AMs are sourced, and the perceived barriers to addressing AMR via One Health approach.

**Methods:**

A phenomenological study design was utilized with applied qualitative research theoretically framed as pragmatism. Twelve in-depth interviews and informal discussions with a One Health focus, were purposively conducted with wide range of stakeholders and community resident of Kapilvastu municipality of Nepal during April 2022. The acquired data was analyzed manually via a thematic framework approach. The study obtained ethical approval from ethical review board of Nepal Health Research Council and University of Leeds.

**Results:**

Nepali and Awadhi languages does not have specific words for AMs or AMR, which is understandable by the community people. Rather, community use full explanatory sentences. People use AMs but have incomplete knowledge about them and they have their own local words for these medicines. The knowledge and usage of AMs across human and animal health is impacted by socio-structural factors, limited Government regulation, inadequate supply of AMs in local government health facilities and the presence of various unregulated health providers that co-exist within the health system. Novel ideas such as the use of visual and smart technology, for instance mobile phones and social media exposure, can enable access to information about AMs and AMR.

**Conclusion:**

This study shows that terminology that is understandable by the community referring to AMs and AMR in Nepali and Awadhi languages does not exist, but full explanatory sentences and colloquial names are used. Despite regular utilisation, communities have incomplete knowledge regarding AMs. Since, knowledge alone cannot improve behaviour, behavioural interventions are required to address AMR via community engagement to co-produce their own solutions.

**Trial registration:**

Not applicable.

**Supplementary Information:**

The online version contains supplementary material available at 10.1186/s12913-023-10470-2.

## Introduction

Antimicrobial resistance (AMR) is the process by which microbes evolve mechanisms to survive the medicines designed to destroy them. AMR is one of the major global health threats of modern times, contributing to 1.27 million human deaths in 2019 alone [1]. AMR is considered a *One Health* challenge as it impacts on the health of humans, animal, plants, and the environment. The evolution of AMR in microbes in a natural process but is exacerbated by exposure to conditions which accelerate resistance such as antimicrobial medicines, climatic changes, and heavy metal pollution [2–5]. As such, AMR is truly a One Health challenge which requires multi-sectoral action. The World Health Organisation (WHO) Global Action Plan on AMR predicts that lower and middle income countries (LMICs) countries such as Nepal are set to bear the highest burden of drug resistant infections in the coming 30 years [6]. This was emphasised by Murray et al.’s work which showed the highest rates of AMR in Sub-Saharan Africa and Southeast Asia [1]. This is due to a combination of factors including population growth, poor water and sanitation hygiene (WASH), and unequal access to healthcare [7–9].

Nepal has seen huge population growth in the past 20 years, putting pressure on healthcare, waste disposal, and food production sectors. The infrastructure of the country lags behind its population boom with pressure on agricultural industries to provide for this growing number of people. Additionally, crowded settlements, a large rural population, recent natural disasters such as the 2015 earthquake, and poor sanitation mean Nepal has a higher prevalence of common diseases than other South-East Asia Region (SEAR) countries [10–12].

Antimicrobial (AM) usage to treat these and other ailments is not always regulated by healthcare providers [13]. In combination, these issues mean AM usage is liberal, for example antibiotics (ABs) may be used to treat simple respiratory symptoms in humans or used for growth promotion in livestock. Future pressures to increase food productivity leading to more intensive farming may drive pressure to use growth promoters more. Such misuse, combined with improper storage and disposal of AMs, is fuelling AMR in both urban and rural regions of Nepal [8, 14].

Nepal is in its early stage of federalization where the new state architecture has three tiers of government – federal government, seven provincial governments and 753 local governments (municipalities), and the power and responsibilities are devolved to local governments [15, 16]. In the new federal structure, health is one of the most decentralized sectors where basic health services fall under the exclusive functions of local government which have the authority to plan, operate, and manage their own health systems, so that health services can be brought closer to homes of residents. This can eventually narrow the gaps in health service access and utilization [15, 16]. However, reports have shown limited institutional capacity to deliver these functions across the government, and lack of clarity and coherence between policies and devolved powers [17].

To successfully tackle the challenge of AMR in Nepal, we must first understand the scale and complexity of the problem. The core issue regarding AMR in Nepal is the very limited information on AMR at community level [18]. Hence, the aim of this paper is to explore local language and terminology used around AMs in the community, commonly used antimicrobials and reasons for their usage, how these antimicrobials are sourced, and the perceived barriers to addressing AMR via One Health approach.

This paper is a part of COSTAR (Community Solutions to AMR) project (2021–2023) [19]. This project seeks to co-create, implement, and robustly evaluate an innovative intervention that addresses the contextual drivers of AMR in Nepal (Kapilvastu) and Bangladesh. COSTAR is an interventional study, where community dialogue approach (CDA) is being co-created and implemented as an intervention in Nepal. This project has multiple components i.e., formative, intervention development, implementation of intervention and evaluation. The results of this formative qualitative analysis will support and inform various components, including intervention of the COSTAR project. This project will be accomplished through the One Health concept, which will rely on an infrastructure for knowledge exchange that will have an impact on national and global policy.

## Methodology

### Research design

A phenomenological study design was carried out in this study with applied qualitative research theoretically framed as pragmatism [20]. The aim of the study is to explore specific themes to contribute to specific policy and practice recommendations as ‘actionable knowledge’ [21].

The methodology of the study is reported in line with the COREQ guidance [22], and a checklist is provided as (Additional file 1).

### Study setting

This study was carried out as a preliminary formative work for the COSTAR project and its study site was Kapilvastu municipality which lies in Lumbini province of Nepal. It has population of around 88,874 with 43,998 males and 44,876 females [23]. This municipality is one of the plain (Terai) region in Mid-Western and shares open border with India. There is geographical, social, and cultural diversity in this municipality, which allows us to explore differences across various diversities.

Furthermore, the healthcare system for human and animal health is based on government and private health care facilities [17]. In Kapilvastu, government facilities include, basic health service centre, health posts, urban health clinic and district hospitals [15, 24, 25]. Similarly, private health facilities include local medicals, pharmacies, clinics, private hospitals whereas quacks and traditional healers are the informal health service providers [15, 24, 25] (Table 1). In the sector of animal health, animal service centre at ward level and veterinary hospital and Livestock Expert Centre at district level are the government service providers whereas agro-vet are the private service providers and lastly, traditional healers are informal service providers [15, 24, 25] (Table 2). Hence, these pluralism in local health system of Kapilvastu municipality, in addition to open border with India could have played an important role in misuse of antimicrobials as many people from this municipality visit the border side of India to seek medical care and over-the-counter medicines at low cost. Further, there is limited information available on the AMR related issues in plain (terai) regions of Nepal [24, 25]. All these observations have been noticed while HERD implemented other health system related project in the same municipality by embedding into the local health system.

**Table 1:** Overview of health facilities Kapilvastu municipality – human health

	Human Health	
Formal	System	Informal health service providers
Government health facilities	Private health facilities	N/A
Health posts	Local medicals	Quack (fake) doctors
Basic health service centre	Pharmacies	Traditional healers
Urban health clinic	Clinics	
District hospitals	Private hospitals	

*Ayurveda is part of formal health system but not included in this table as it is not directly linked with AMR

**Table 2:** Overview of health facilities Kapilvastu municipality – animal health

	Animal Health	
Formal	System	Informal health service providers
Government health facilities	Private health facilities	N/A
Animal service centre at ward level	Agro-vet	Traditional healers
Veterinary hospital and Livestock Expert Centre at district level		

### Data collection

The research team purposively conducted ten qualitative interviews and informal discussions with 12 adult participants. All were community residents and represented a range of stakeholders from one health perspectives (doctors, vets, etc.) within the local health system. People who did not provide consent and of age less than 18 years were not included in the study. To identify and purposively select the appropriate participants for the study, the research team coordinated with stakeholders from the health section of Kapilvastu Municipality and District Animal Health Office. Interviews were conducted with those who provided written informed consent by using guide which had separate set of questions for participants from different domains: human health, animal health and community members.

The number of interviews were determined based on saturation of data during the time of data collection. The duration of the interviews ranged from 30 min to one hour. Data collection was done in April 2022. Characteristics of participants are presented in Table 3.

**Table 3:** Characteristics of study participants

Participant ID	Occupation /role	Gender	Area	Service provider
1	Owner of local agro-vet	M	Urban	Agro-vet
2	Owner of local agro-vet	M	Rural	Agro-vet
3	Local pharmacist	M	Urban	Pharmacy
4	Local pharmacist (medical)	M	Rural	Pharmacy
5	Qualified doctor from private clinic	M	Urban	Private clinic
6	Health post in-charge (health assistant)	M	Urban	Health post
7	Health post in-charge (health assistant)	M	Rural	Health post
8	Chief of Veterinary Hospital and Livestock Service Expert Centre	M	Urban	Veterinary Hospital
9	Community resident	F	Urban	N/A
10	Community resident	F	Urban	N/A
11	Community resident	M	Rural	N/A
12	Female community health volunteer (FCHV)	F	Urban	N/A
	**Total participants: 12**			

The data were digitally recorded after securing the participants’ written consent. In addition to the recording, the research team (AP and BB) took field notes in Nepali that captured observations and informal discussions during the data collection period. All the interviews were conducted in Nepali. They were transcribed and then translated to English by well-trained translators fluent in Nepali and English, adhering to the transcription/translation guidelines of the organization, under the supervision of the core research team. For quality assurance, four translations and transcriptions were checked against the transcripts and audios respectively and corrections were made as needed.

### Ethics

For this study ethical approval was obtained from Nepal Health Research Council (NHRC) with reference number 3098 Ethical Review Board (ERB) protocol registration number 189/2021. The study also obtained approval from the University of Leeds, Faculty of Medicine, and the Health Ethical approval Board in February 2020 under the project name: MREC 20–034 – ‘Engaging communities to address antimicrobial resistance: Identifying contextualised and sustainable community-led solutions in low resource settings’, which was later named COSTAR.

### Data analysis

The research team developed a codes, using both *a priori* and emergent codes in the analysis process. First, researchers (AP, BB and AA) generated an *a priori* code based on the objectives of the study and the questions in the interview schedule. Additionally, another researcher (LG) conducted a manual, thematic analysis of the coded interview transcripts [26] that enabled them to identify key sub-categories (subthemes) and categories (themes). Additional researchers (JM, SB) contributed to the data analysis process by offering suggestions on refining the themes and subthemes in iterative stages to generate clear and concise findings. The key themes and sub-themes generated via the data analysis are summarised in Table 4.

**Table 4:** Key themes and sub-themes

Themes	Subthemes
**I – Commonly used AMs in human and animal health**	Antibiotics (AB) and Antimicrobials (AM) Local terminology used by community to refer antimicrobials
**II – General practice of consuming AMs in the community**	Socio-structural factors Knowledge and practice of AM usage Social media information and visual aids
**III - Preliminary assessment of AMR drivers**	Misuse and overuse of AM Lack of regulatory mechanisms and policies Lack of AMR awareness and AM disposal behaviours

## Results

It is important to state that the literal Nepali and Awadhi languages translation of the terms ‘antimicrobial’ and ‘antimicrobial resistance’ were not understood by the community, but rather a full sentence must be used to explain each term. The research team asked questions focused on ‘antimicrobials’ but the participants and service providers answered mostly referring to ‘antibiotics’. Hence, this is an important consideration and finding of our study. Additionally, antibiotics were the most identified type of antimicrobials during the conversations with community members. As such, data collectors often used the translational sentence for antibiotics as a way of supporting community members to understand the term antimicrobial. For these reasons, the findings section does discuss antibiotics specifically in many areas.

### Theme I - Commonly used antimicrobials in human and animal health

#### Antibiotics (AB) and antimicrobials (AM)

Service providers mentioned a range of commonly used antimicrobials (AM), particularly antibiotics, in the human and animal health systems (Tables 5 and 6). However, in this qualitative study we did not aim to quantify their use, but to explore the community’s own understanding of these medicines.

To contextualize the relative importance of these drugs, classification of antibiotic medicines is explained in Tables 5 and 6 with reference of WHO AWaRe classification [27]. All the drugs identified in Tables 5 and 6 are also mentioned in Nepal’s National List of Essential Medicines released by the [28] which means that they are regulated medicines by the Nepal Government and used by the service providers and the population.

**Table 5:** Commonly used antimicrobials (generic names) in human health identified by this study

No.	Common medications used in human health	WHO list classification of medicines
1	Cefixime	Watch
2	Amoxicillin	Access
3	Metronidazole	Access
4	Ciprofloxacin	Watch
5	Azithromycin	Watch
6	Sulfadiazine/trimethoprim (Cotrimoxazole) -	Access
7	Fluconazole (antifungal, not AB)	**NA – antifungal not antibiotic**

**Table 6:** Commonly used antimicrobials (generic names) in animal health identified by this study

No.	Common medications used in animal health	WHO list classification of medicines
1	Streptomycin	Watch
2	Amoxicillin	Access
3	Tetracycline	Watch
4	Ampicillin	Access
5	Cloxacillin	Access
6	Oxytetracycline	Watch
7	Gentamicin	Access
8	Ceftriazone	Watch
9	Penicillin	Access
10	Cypermethrine (commonly used for external parasitic infection in animal, it is not AB)	**NA – insecticide not antibiotic**

Health service providers suggested that in most of the cases, drugs used to treat humans and animals are same by their generic names but differ by dosages. However, the dosage might vary as per the species of animals and some medicines also differ by their trade name.

*“Some antibiotics which are used for human health problems, these medicines are also used in veterinary medicine with different doses and names. For example: penicillin is used in the dose of 4 lakhs to 5 lakhs (4 to 5 hundred thousand units = 250–313 mg) in human while for animals, doses of 20 lakhs to 40 lakhs (20 to 40 hundred thousand units = 1250–2500 mg) are used.” (P 2 – owner of local agro-vet).*

They reported that Cefixime is the most used AB in the community.

*“Cefixime is the cheapest antibiotic available in the market at present condition. [It is] the mostly used and misused antibiotic.”* (P 5 - private clinic doctor).

Similarly, antifungal drug Fluconazole, are reportedly misused by the community due to their low cost. People understand Fluconazole as a medicine taken every week, which is cheaper than specific antifungal treatments.

*“People go to pharmacy and ask for medicine for rashes with 50 rupees. It is also not the fault of pharmacist because a person may come with 50 rupees and ask for the anti- fungal drugs when other anti- fungal drugs are not found in 50 rupees. Fluconazole is the only drug which comes at 50 rupees.”* (P 5 - private clinic doctor).

There are several concerns about the use of AMs. Commonly used AMs in this region are similar across human and animal sectors. This can allow resistant infections to develop and spread between species. In terms of framing community engagement messages, it is important to consider access to medicines and what is a realistic alternative for both providers and users. Focusing messages on prudent use of AMs for sick animals can limit AM use and the risk of AMR developing and spreading between species. It is promising that none of the ABs discussed here were on the WHO’s *Reserve* category of drugs, which should be protected for the treatment of multidrug resistant infections in humans.

#### Local terminology used by community to refer to antimicrobials

Community were not able to understand the literal Nepali and Awadhi translation of the word ‘antimicrobials’ and ‘antimicrobials resistance’. Hence it was essential to explore the local terminology used in the community when people seek health care services. The medicines listed in (Table 7) are different forms of AMs which are commonly understood by various colloquial names in the community. Different health workers from human health explained during interviews that community members do not understand the term ‘antimicrobial’ or ‘antibiotic’ but they usually refer to these groups of medicines with the specific local terminologies, and they think they should be used whenever they experience specific symptoms (Table 7).

**Table 7:** Local terminology used in community health seeking behaviour

Health issue identified	Type of medicine used	Local terminology used
Dysentery, diarrhea	Metronidazole, antiprotozoal	*metro*
Fever	Amoxicillin	*amoxy*
Typhoid	Ciprofloxacin	*cipro*
‘when we get severely sick’	Antibiotics	*- high dose* *- medicine of power*

*“People ask for medicines of high dose if they want antibiotics. Some people ask for medicines of power which means antibiotics.”* (P 5 – private clinic doctor).

Findings show that the terminology used locally for AMs is strongly impacted by the level of education and the type of health centre community members visit when they seek health support. If they go to a local health post to seek treatment, they tend to ask for specifically named medicines as shown in Table 7. But if they go to clinics or private hospitals, it has been reported that they do not demand medicines or AMs when they see a doctor.

*“Hill originated Brahmin/Chhetri people residing in Terai are more educated in comparison to other terai people*. *People other than hill originated Brahmin/Chhetri, directly ask for strong medicine, injection (antibiotics) etc. Hill originated Brahmin/Chhetri ask for medicine with low dose at first and if it does not work, then only, they will seek medicines of higher doses (antibiotics).”* (P 5 – private clinic doctor).

Furthermore, the level of education and literacy that people have in this region has an impact on their health-seeking behaviour and knowledge of medicines such as AMs. Being able to read the name of the medicine and the required dose is difficult for those with low literacy levels and memorising such technical language is also not easy, hence the support of the health staff seems to be crucial in this case.

*“Educated people can read the label, so they can understand but uneducated people may not know about anti-fungal, anti-helminthic medicines. They may understand the medicine but they may not know the name of the medicine.”* (P 3 – local pharmacist).

The findings stress that there exists some differences in educational status of people belonging to different ethnicities. However, religion and ethnicity alone have a minimum role to play in influencing AM knowledge. Geographically, people belonging to similar culture and mindsets live together in the same community. People living in same community share common culture, social norms and values, beliefs and thoughts. Hence, it is rather the education and the community they live in contribute to shaping behaviours towards AM:

*“There is not much difference in terms of religions and ethnicity but there are many differences in terms of education. For example: in ward no. x, people are educated with higher level degrees in terms of qualification. There, people consult with doctors before using antibiotics. Practice of people depends on education and the environment, but it does not depend on religion and ethnicity.”* (P 5 - private clinic doctor).

Our findings show that Kapilvastu community members have their own working terminology for different medicines that fall under the category of AMs. They also recognise several common AB and antifungal medicines by name, but they do not know the word ‘antibiotic’ or ‘antimicrobials’. However, they have been using these medicines. This has implications for the design of AMR interventions aimed at engaging with the community, as it suggests a working knowledge of antimicrobials is present but incomplete.

Anecdotal conversations with an agro-vet reveal that in the case of agriculture, insecticides and pesticides are commonly used. However, as discussions progressed, the use of *insecticide* appeared to refer to the treatment of viral and bacterial infections by spraying streptomycin, which is actually an antibiotic. Indeed, agro-vets struggled to name AMs other than ‘streptomycin’ suggesting that knowledge of AMs in this sector is limited and could be driving misuse. This also shows misunderstanding of terminologies and drugs among service providers as well.

### Theme II - General practice of consuming AMs

#### Health behaviour and socio-structural factors

The health-seeking behaviour for human health in the community starts with visiting the nearest health services available in the area. The findings highlight that this is impacted by the level of education and knowledge people have, their age and their geographical setting. For instance, participants reported that those who are educated and live in urban areas go directly to the hospital, while those living in rural areas go to a medical store first:

*“The reason for these differences in priorities of health services is due to education. Here [urban], people are educated so they don’t go to medical store directly while they face health problem. They go to hospital first. In village areas, they don’t have much knowledge, so they go to hospital only at the last stage.”* (P 12 - Female community health volunteer).

Also, knowledge of AMs is more associated with young people who are assumed by the community to be more educated. This is likely to be aided by more information available on mobile phones and young people being thought to be more likely and able to search for such information.

When engaging in AM usage behaviour, people also consider ‘money and distance’ to the health post. For these reasons, in rural areas, they tend to first purchase and use ABs from a pharmacy or medical which is closer to their homes and if this does not work, then they visit a nearby health centre for assessment and further treatment.

*“People take antibiotics first and if antibiotics do not work, then they come here [health facilities]. There is a medical (drug store with or without checkup facility) in every two villages…. There are two factors like money and distance. To save these factors, people first visit the local medical store and if it does not work there, then only they come to our clinic.”* (P 5 –private clinic doctor).

The health behaviour in the community is, to some extent, also impacted by people’s beliefs in traditional healers which are part of the informal health system. Traditional healing is the practice of culturally and spiritually healing illness via *dhami/jhankris* and *gurus* [29].

*“People also have religious belief. They go for traditional healing along with medical practices.”* (P 5, urban clinic doctor).

Similarly, people make use of the informal services from ‘quack’ (fake) doctors, especially in the border area with India. Community members informally termed them as ‘Bangali doctors’ as they were originally from the state of West Bengal, India.

*“Like in the border area, there are availability of quack doctor as well. These doctors give injections which contains steroids, so it works a little faster, so people have high trust on them.”* (P 6 – health assistant).

Participants also reported that in case of minor health issues they tend to use home remedies, consume fresh food and fruits, and ensure a clean-living environment This suggests that they tend to follow a pluralistic health system including the use of home remedies and non-medical prescriptions.

Furthermore, structural factors at the level of supply in the local government health facility, such as not having enough stock to provide patients with a full dose, impact on peoples’ AM usage, which then leads to community members not completing a full AM dose. It is important to specify that the lack of stock was an issue specific to government health facilities, which provides AM to people free of cost.

*“People come and we give them the dose for three days. We also have to look at the stock. We cannot give medicine dose for seven days because we have to give medicines for other people as well.”* ( P2 – health worker at the health post).

For animal health, there are limited facilities to carry out culture tests on animals. Furthermore, service providers and community members mentioned that, in a place like Nepal, where people cannot afford health services for themselves, it is not feasible to carry out culture and antibiotic sensitivity tests for animals and the long waiting time for a test result may out-turn the death of an animal.

*“We do not have any tests. We do not have X- ray service, ultrasound, pathology service, etc. The diagnosis all depends on our prediction. An antibiotic is prescribed in a village, and if that antibiotic does not work, then the animal is taken to the market or Taulihawa district hospital (district level veterinary hospital and Livestock Expert Centre).”* (P 1 – owner of local agro-vet).

#### Differences between knowledge and practice

In the case of human health, some of the community members mentioned they are aware of the importance of taking a full dose of AMs and they practice it accordingly, by following the advice of the doctor although they may not know the names of the medicines. They also share their knowledge with others in the rural community to take the complete AM dose as prescribed.

*“I do not leave any medicines. I take medicines to full doses. I teach other people as well about the importance of taking full course of antibiotics.”* (P 10 – rural community member).

However, positive individual practices do not appear to translate into norms at the collective level, as participants reported different patterns in community practices on AM use and not being able to get a complete dose from health providers.

*“I cannot say the same about other people in this community. They go to the clinic or pharmacy and tell about their illness. They consume the medicine provided by the pharmacist. If the medicine has proven to work for them, they visit the pharmacy next time when they get sick and ask for it again. Sometimes, they also tell the pharmacy: “please give me the medicine for two days as my illness got cured in two days last time, so no need for a complete course”. And the pharmacy also complies with their demands.”* (P 10 – rural community member).

This quote suggests that it may be difficult for the pharmacist to sell a full course, particularly if the patient has experience of a short dose working before. Hence it becomes difficult to change these learnt behaviours and also it may not be clear if all pharmacists have the knowledge required to do that. However, assessing the knowledge of pharmacists or health workers was beyond the objectives of this study.

Similarly, in agriculture and animal husbandry individuals tend to purchase a short course of AMs rather than completing a full dose. Thus, clear similarities appear between the (mis)use of AMs in human and animal health.

“*Yes, I tell them about the required doses of antibiotics, but they do not take the required doses. They just take one to two doses of medicines and if necessary, then next day, they come to take medicines. Many people do not take medicines after one to two doses. Very less people take the full doses of medicines*” (P 1 – owner of local agro-vet).

One of the agro-vet participants reported that the Government has been providing training on good agriculture and animal husbandry practice to the farmers. However, trainings are reportedly scarce and considered inadequate since only those who are licensed to carry out animal husbandry or agriculture can participate in such events. This means that those who want to participate may not be able to join the session because of the eligibility criteria (i.e. need to be licensed). The agro-vet reported that people take part in these trainings mostly for financial purpose, to get the allowance provided by the Government. On the other hand, those who get the chance to participate may not be interested in it.

*“There are not many trainings available. Some trainings are organized once or twice in a year, and when trainings are organized, we just go to the program to take allowance. We just do some introduction and take little information.”* (P 2 – owner of local agro-vet).

#### Social media information and visual aids

Service providers, particularly health workers from health post and agro-vet, shared their observations about how community members use smart technology, namely mobile phones to facilitate conversation with health professionals for example by taking pictures of medicines.

“*Most of the people do not have enough information about antibiotics but they have mobile phone. So, they click the pictures of medicines which they are using and bring the pictures to us [veterinary practice] for medicines.”* (P 1 – owner of local agro-vet).

Our findings point to novel ideas such as the use of technology, namely mobile phones to counteract the lack of awareness of AM usage impacted by the structural factors discussed earlier. Via their phones, young people have access to the internet and social media sites, such as YouTube, regardless of their social status. Such social media exposure can enable access to information about AM usage.

Similarly, when researchers asked the participants for suggestions on how to engage with community members in further studies, and how to ask questions about ABs in a way that could be understood at the community level, respondents pointed to potential future activities supported by visual aids. They suggested, for example, showing pictures of commonly used medicine packages to community members to help them better engage in a discussion about the usage of AM.

### Theme III - preliminary assessment of AMR drivers

#### The misuse and over-use of AMs

Participants reported regular misuse of AMs, specifically ABs, in the community as a key driver of AMR in both human and animal health. This behaviour is driven by the community members’ wish to recover fast from an illness and by the practices of health staff who give ABs on demand in an attempt to cure patients faster.

*“In the case of private medical store, people also demand antibiotics by themselves. Medical personnel also usually give antibiotics to solve the health problems faster.” (P1 6 – health worker)*.

Directly linked with the misuse of ABs is their availability without prescription which may lead to over-use. This was amplified during the Covid-19 pandemic, when one type of AB was prescribed for Covid-19 related symptoms. A clear example of misuse as COVID-19 is viral, not a bacterial infection.

*“There is availability of antibiotics everywhere. Anyone is giving and prescribing antibiotic. In COVID-19, Ceftriaxone was used so widely in this area that this medicine became scarce. Ceftriaxone costs 35 rupees but during COVID-19, Ceftriaxone was not found even at 100 rupees.”* (P 5 – urban clinic doctor).

In case of animal health, fast growth of poultry and other animals that encourages quick financial gains, have been reported as one of the key reasons for AMR in the community. This type of behaviour is common regardless of knowledge about the negative consequences that it entails.

People are giving hormonal medicines to chickens to raise them faster which have consequently negative effects on ourselves.

“*Now-a-days, farmers have also been educated. They have studied poultry, fisheries, etc. But there is misuse of antibiotics as well.*” (P 2 – owner of local agro-vet).

#### Lack of regulatory mechanisms and policies on AMR

According to the stakeholders, such as official from district animal health office, private clinic doctors and agro-vet staff, the use of AMs and specifically ABs is not sufficiently regulated at the national level, hence community members can purchase and misuse AMs, as they are easily available.

“*There are no proper policies about who can use the antibiotics and who cannot. Antibiotics are available everywhere and anyone is using antibiotic randomly without standard treatment protocol.”* (P 5 –private clinic doctor).

The lack of enforced law mechanisms for AM usage also applies to animal health and agriculture. Findings suggest that community members working in this sphere engage in commerce with medicines that are not subject to sufficient quality checks and registered health practices. This is an important point also in the context of the geographical border with India which allows for people and goods to move easily between the two countries.

“*People also go to border of India to buy medicines. …People also use unregistered medicines. Some people sell medicines which are not registered by Department of Drug Division. People who are selling agricultural products are also selling medicines which belong to unregistered medical practices. There are not much quality control and regulation mechanism.”* (P 2 – owner of local agro-vet).

Furthermore participants reported misuse of licenses by people who do not have the required education and whose misconduct for business purposes has not been penalized. Hence stricter law enforcement in this domain has been strongly recommended.

*“Many people who have not even studied one word are also running veterinary clinic by misusing the license of other people. People have done business of crores (millions) without having authority. Authorized organizations of government should check and measure such misconducts.”* (P 2 – owner of local agro-vet).

#### Lack of awareness about AMR and AM disposal behaviours at community level

Disposal of AMs has often been related to a lack of awareness, about AMR and appropriate use of AMs in human and animal health.

*“People are not educated and aware about antibiotics and antibiotic resistance. People do not even know that they are using antibiotics randomly… It is not their fault.”* (P 5 –private clinic doctor).

The disposal of AMs is not regulated and not penalised, hence there is no specific reported practice followed at the community level. Some people and pharmacies dispose waste medicines in garbage collection sites or dustbin or open spaces.

*“In this community, wastes such as (used) sanitary pads, and medical wastes are thrown in an open area. The pharmacy across this road throws waste quite carelessly in the open environment. They are careless because they are not aware of the adverse consequences of such practices.”* (P 11 – community resident).

In terms of animal health, participants highlighted that some of the agro-vets take back the remaining medicines such as ABs, vitamins and other supplementary medicines that come in solid form if they are returned with the payment of bills, which suggests positive behaviour.

## Discussion

This study explored local terminology used in the community to refer antimicrobials including antibiotics. Although community people did not always understand the term ‘antimicrobials’ or ‘antibiotics’ they did utilize a variety of colloquial words regarding drug use linked to specific symptoms. This contextual recognition of medication is similar were the findings of another study conducted in Congo [30]. Health seeking behaviours of community people are constrained by various socio-structural factors such as low physician to patient ratios (1:1724), lack of supply in government health facilities, lack of affordability of healthcare services, as shown in other Nepal-based studies [10, 31]. These factors indicate that most people in Nepal rely on other service providers like, drug dispensers (medical and pharmacies), quacks and traditional practitioners [10, 32], which is also evidenced by our study.

This pluralism in health system have been one of the reasons for inappropriate use of antimicrobials in Nepal [25]. For example, in community practice of both human and animal health sectors, prescription is not required to dispense AMs in Nepal and this can promote inappropriate use of AMs, as evidenced in our study. The wider literature clearly shows that AMR is driven by complex intersections around accessibility and availability of AMs, especially ABs. This includes over-the-counter (OTC) and prescription sales, irrational use, failure to follow the prescribed course of ABs as well as a lack of an effective AMR surveillance [7, 24, 25, 33]. Other studies conducted in Nepal indicate that animal health experts have little control over AMs and there are limited surveillance strategies and data to capture the AMs use and AMR risks in Nepal [34]. Hence, co-existence of a pluralism in health system of Nepal and other countries calls for greater collaboration between the formal and informal healing systems in order to promote appropriate use of AMs by regulating standard treatment protocol [24, 25, 27].

Furthermore, our study indicates that the education and location (urban/rural) of people influenced their knowledge and practice of AMs usage. This is widely supported by other research conducted in Nepal [33]. In addition, education and awareness about AMs use varied among different ethnic groups residing in Kapilvastu municipality. For example, hill-originating Brahmin/Chhetri were more aware in comparison to other ethnic groups residing in the same area. This variation in educational status is supported by further analysis of National Demographic Health Survey, Nepal [35]. This study shows that lack of awareness about AM and AMR at the community level, including both consumer and supplier is also related to inappropriate disposal practice of AMs.

It is recognized throughout the literature that knowledge alone is often not enough to change behaviours in general [24], and specifically around AM use and AMR in low resource settings. Reports show that District Drug Administration of Nepal conducts several AMR activities around personnel training and drug quality surveillance in Nepal [28]. For instance, they developed a community pharmacy training module focused on drug prescription and distribution for community pharmacy which includes an AMR awareness component [17]. However, our study shows that, in practice, community drug dispensers do not dispense complete dose of AMs to the people due to different types of structural barriers (such as people’s inability to purchase full course of drugs, lack of awareness about need to purchase full course).

This practice is further followed by limited specific regulations that prevent community members from buying AMs over the counter. Literature suggests, practice of drug dispensers in Nepal is largely unregulated and they have poor compliance with good pharmacy practice [24]. This study have similar findings in case of animal health. Literature suggests, there is no active surveillance in the animal health sector in Nepal [17]. Moreover, there is easy import of food, animals and meat from the open border with India and there is no specific mechanism for testing these products for AMR [17].

## Conclusions

This study shows that the local term for AMs and AMR in Nepali and Awadhi languages does not exist, but full explanatory sentences and nick names for different AMs are used. People have been using it but have incomplete knowledge about AMs. The knowledge and usage of AMs across human and animal health is impacted by socio-structural factors, lack of government regulation and lack of supply in local government health facilities and the challenges of various unregulated health providers that co-exist within the health system.

Despite the progress made in improving AMR awareness at the policy and global level and via National Actions Plans in LMICs, critical gaps remain [36]. Hence, a different more creative approach might be required to tackle this challenge beyond knowledge provision. Using social media for raising awareness on AMR in the context of increased use of mobiles in LMICs is an opportunity for dissemination of information that is faster and cheaper than traditional methods and can reach larger audiences in short period of time [37]. This could be taken further, for instance by creating online games with a learning objective on AMR for school children [37].

Furthermore, behaviour change is a key objective of the global action plan on AMR, but many countries lack the support needed to achieve this objective. There is a lack of academic research on AMR in Nepal regarding the behavioural interventions that can help address the problem [17]. However, it has been acknowledged that awareness-raising approaches alone at community level are not sufficient to create meaningful behaviour change on issues such as inappropriate use of AMs [38–40]. Thus, communities must be engaged and encouraged to co-produce their own solutions [38–40]. Our formative qualitative study is an important tool that contributes to filling in this gap by providing contextual information on AMs use and AMR drivers. This then lays the ground for developing AMR interventions co-produced by communities to achieve behavioural changes.

### Limitations

In this study we were not able to purposively sample certain types of people who might have provided useful perspectives on our topic, especially the ‘quack’ doctors and traditional healers. However, the participants spoke about them during the interviews, adding to the richness of our findings and showing the importance and the challenges of the informal, unregulated health system in Nepal, which currently co-exists as a part of the healthcare system.

We are also aware and we reported that the terms AB, AM and AMR could not be directly translated from English into the local languages due to the linguistic differences. The term ‘antimicrobials’ was not clearly conceptually differentiated by participants from the local pharmacies and agro-vet in most cases. Nonetheless, they have used antifungals to treat various related infections. Furthermore, the term antimicrobial is not as common as the term antibiotic in the community. Hence, most of the transcript quotes refer to antibiotics.

### Electronic supplementary material

Below is the link to the electronic supplementary material.

Supplementary Material 1

Supplementary Material 2

## Data Availability

All data generated or analysed during this study are included in this published article and its additional file 2.

## Abbreviations

ABsL: Antibiotics
AMs: Antimicrobials
AMR: Antimicrobial resistance
COSTAR: Community solutions to antimicrobial resistance
ERB: Ethical review board
FCHVs: Female community health volunteer
LMICs: Lower- and middle-income countries
NHRC: Nepal Health Research Council
OTC: Over the counter
SEAR: South-East Asia Region
WASH: Water sanitation and hygiene
WHO: World Health Organization
