# Supplementary material for: Exploring community insights on antimicrobial resistance in Nepal: a formative qualitative study
Source: BMC Health Serv Res. 2024 Jan 11;24:57. doi: 10.1186/s12913-023-10470-2 (PMC10782613; doi:10.1186/s12913-023-10470-2)
Supplement: Supplementary file 1 — Supplementary Material 1 [file 12913_2023_10470_MOESM1_ESM.docx]

**Table 1. Consolidated criteria for reporting qualitative studies (COREQ): 32-item checklist applied to the paper.**

| **No** | **Item** | **Guide questions** | **Answers applied to the paper** |
| --- | --- | --- | --- |
|  |  | **Domain 1: Research team and reflexivity** |  |
|  | **Personal characteristics** |  |  |
| 1 | Interviewer | *Which author/s conducted the interview?* | AP and BB |
| 2 | Credentials | *What were the researcher’s credentials?* | AP – Master's in Public Health; Bachelor of Science in Nursing  BB – Master's in Nutrition and Dietetics, Bachelor's in Nutrition and Dietetics |
| 3 | Occupation | *What was their occupation at the time of the study?* | AP – Senior Research Officer at HERDi;  BB – Intervention Coordinator for the Field Office at HERDi |
| 4 | Gender | *Was the researcher male or female?* | AP – female; BB – male. |
| 5 | Experience and training | *What experience or training did the researcher have?* | AP – Researcher with more than 4 years of experience in different participatory qualitative approaches (community engagement, participatory action research, participatory videos, photo voice, in-depth and key informant interviews, focussed group discussions), conducted interviews for this study.  BB – Researcher with more than 3 years of experience in research and trained in conducting qualitative interviews, collected data for this study. |
|  | **Relationship with participants** |  |  |
| 6 | Relationship established | *Was a relationship established prior to study commencement?* | No relationship with the participants was established before the commencement of the study. |
| 7 | Participant knowledge of the interviewer | *What did the participants know about the researcher? e.g. personal goals, reasons for doing the research?* | Participants did not know any of the researchers.  Researchers were introduced to the participants only during the data collection period. However, all participants were explained about the purpose of study and researchers’ visit to their place for data collection during written informed consent process. |
| 8 | Interviewer characteristics | *What characteristics were reported about the interviewer/facilitator? e.g. Bias, assumptions, reasons and interests in the research topic* | Two members of the research team conducted interviews with participants.  AP is a registered nurse and public health professional, working as a researcher at HERD International. She has experience of conducting qualitative research using different approaches for more than 4 years in different health topics.  BB is nutritionist and a researcher at HERD International working in health-related projects. He has been trained to conduct qualitative interviews and focussed group discussions. |
|  |  | **Domain 2: Study design** |  |
|  | **Theoretical framework** |  |  |
| 9 | Methodological orientation and Theory | *What methodological orientation was stated to underpin the study?* | This is applied qualitative research framed theoretically as pragmatism (Feilzer 2010). |
|  | **Participant selection** |  |  |
| 10 | Sampling | *How were participants selected?* *Purposive, convenience sampling? Or snowballing?* | Purposive sampling |
| 11 | Method of approach | *How were participants approached? e.g. face-to-face, telephone, mail, email* | Participants were approached via face-to-face coordination meeting prior to the data collection. Data collection was done via face-to-face interviews. |
| 12 | Sample size | *How many participants were in the study?* | 12 participants |
| 13 | Non-participation | *How many people refused to participate or dropped out? Reasons?* | None of them refused to participate in the study. |
| 14 | Setting of data collection | *Where was the data collected? e.g. home, clinic, workplace* | Data collection was done at the participants’ workplace as per their convenient time. However, few interviews with community members were conducted at their home as per their given appointment. |
| 15 | Presence of non-participants | *Was anyone else present besides the participants and researchers?* | In most of the cases, interviews were conducted with participants alone. However, in some cases, participants (especially community members) took help from their family members to answer the questions about the health seeking behaviour of their family members. |
| 16 | Description of sample | *What are the important characteristics of the sample? e.g. demographic data, date* | Occupation, geographic area (urban / rural) and the places where they provide services. |
|  | **Data collection** |  |  |
| 17 | Interview guide | *Were questions, prompts, guides provided by the authors? Was it pilot tested?* | Yes.  Yes. |
| 18 | Repeat interviews | *Were repeat interviews carried out? If yes, how many?* | All of the interviews were completed during a single attempt. Hence, repeat interviews were not required. |
| 19 | Audio/visual recording | *Did the research use audio or visual recording to collect the data?* | Yes, audio recording. |
| 20 | Field notes | *Were field notes made during and/or after the interview or focus group?* | Yes, during and after the interviews. |
| 21 | Duration | *What was the duration of the interviews?* | The duration of the interviews ranged from 30 minutes to one hour |
| 22 | Data saturation | *Was data saturation discussed?* | Yes |
| 23 | Transcripts returned | *Were transcripts returned to participants for comment and/or correction?* | No |
|  |  | **Domain 3. Analysis and findings** |  |
|  | **Data analysis** |  |  |
| 24 | Number of data coders | *How many data coders coded the data?* | Four researchers: AP, BB, AA and LG. |
| 25 | Description of the coding tree | *Did authors provide a description of the coding tree?* | No, the analysis was done manually. |
| 26 | Derivation of themes | *Were themes identified in advance or derived from the data?* | They were mixed, three coders identified themes in advance based on the interview questions and a fourth coder identified themes derived from the data. Other researchers and co-authors checked the themes and refined them. |
| 27 | Software | *What software, if applicable, was used to manage the data?* | Data were managed manually using MS Excel and MS Word. |
| 28 | Participant checking | *Did participants provide feedback on the findings?* | No, participants did not provide feedback on the findings. |
|  | **Reporting** |  |  |
| 29 | Quotations presented | *Were participant quotations presented to illustrate the themes / findings? Was each quotation identified? e.g. participant number* | Yes.  Yes. |
| 30 | Data and findings consistent | *Was there consistency between the data presented and the findings?* | Yes. |
| 31 | Clarity of major themes | *Were major themes clearly presented in the findings?* | Yes. |
| 32 | Clarity of minor themes | *Is there a description of diverse cases or discussion of minor themes?* | Yes. |

Source: Tong et al. (2007), ‘Consolidated criteria for reporting qualitative research (COREQ): a 32-item checklist for interviews and focus groups’, *International Journal for Quality in Health Care*, Volume 19, Issue 6, December 2007, Pages 349–357, <https://doi.org/10.1093/intqhc/mzm042>
