# Supplementary material for: Exploring community insights on antimicrobial resistance in Nepal: a formative qualitative study
Source: BMC Health Serv Res. 2024 Jan 11;24:57. doi: 10.1186/s12913-023-10470-2 (PMC10782613; doi:10.1186/s12913-023-10470-2)
Supplement: Supplementary file 2 — Supplementary Material 2 [file 12913_2023_10470_MOESM2_ESM.docx]

**Supplementary material 2 - Interview transcripts**

**Interview Transcript 1**

Interview details

| Participant ID | 1 |
| --- | --- |
| Occupation / role | Owner of local agro-vet |
| Gender | M |
| Area | Urban |
| Service provider | Agro-vet |

Index:

I: Interviewer

P: Participant

I- “Namaste! “

P- “Namaste ma’am!”

I- What are the main problems with which people visit this place?

P- This is an agro vet. So, people come here with the problems of their animals and birds like fever, wounds, cuts, bruises, foul pox, =smallpox=, =smallpox=, =[ranikhet]= in chicken, =FMD= [Foot and Mouth Diseases] in cow, buffalo, rabies in bog bite, vyagute, charchare, Hemorrhagic Septicemia and Blood Quarter [HSBQ], fever, wounds in mouth, eczema, etc. in animals. Around 80% of the problems of animals are being solved by medicines of veterinary. Some antibiotics which are used for human health problems, these medicines are also used in veterinary with different doses and names. For example: penicillin is used in the dose of 4 lakhs to 5 lakhs in human while for animals, doses of 20 lakhs to 40 lakhs are used. Other medicines used are =oxytetracyclin=, =tetracyclin=, =procaine penicillin=, =ampicillin cloxacillin=, =norfloxacin=, =zentamycin=, =ceftriaxone= etc. For human, =ceftriaxone= is used for 500 mg while for animals, some antibiotics are also produced from same company and same producer for both humans and animals.

[1:55] I- How much antibiotics are used in agriculture sector?

P- My shop is 90% about veterinary and 10% about agriculture. Boys at the side of my store run shops for agricultural products. I am not much into agricultural products. Less people come to my store for agricultural products. Most of the people visit me for veterinary problems and for their solutions. There are antibiotics for agriculture as well. For example: streptomycin of 6 grams to kill bacteria.

I-How many years have you been working in this agro vet?

P- My agro vet store is the oldest store in =Kapilbastu= district. My brother is the founder of this agro vet store. When I was 16 years old, I studied ISC. Ag [Indian School Certificate- Agriculture] in India and came back to Nepal to run this agro vet store. I have worked in this field for 36 years. My brother took training for JTA [Junior Technical Assistant- Agriculture] in =New Baneshwor= and started this store. [3:39] There was not much veterinary production and veterinary diseases. There were only few medical for human health problems. I have 6 brothers. We ran this shop with a small investment. I have done my living earning from this tore today. I have a good income at present condition. I run my family with the income of this store.

I-When people see problems in their domestic animals, which is the first place where they go?

P- [5:3] There are only 2 to 4 agro vet in market at present condition. People come to our shop when they have problems in their animals at first. People come to my shop because I am running this shop since a very long period of time. We have few agro vet shop keepers who are running from long period of time. I have about 40 to 50 customers every day.

I-When people come to your shop, what type of complains do they bring?

P-They don’t know about bacteria, antibiotics, virus etc. They say that have have diseases, wounds, bruises etc. Now-a-days, farmers have also been educated. They have studied poultry, fisheries etc. But, there are misuse of antibiotics as well. My neighborhood brother of agro vet shop also gives many antibiotics which are more than necessary. It is about business for them. But I can’t do it. It does not fall under my ethics.

I- That means, antibiotics can be bought over the counter?

P- I have got the education so that I can’t go against my ethics. I give medicines for pain, bruises, fever, cuts, and if necessary. I give simple antibiotics like tetracyclin, cotrimoxazolle etc. Other specific antibiotics should be prescribed by doctor. I suggest the farmers to go to hospital and bring the paper of prescribed medicines but sometimes, it becomes difficult for them to go to hospital due to lack of money, time etc. So, they request me to give them medicines. They don’t want me to return them without medicines. I also give antibiotics but for lactin period, I don’t give antibiotic carelessly. In lactin period, if antibiotic is given to cattle, it reduces their milk production. [7:25] During pregnancy, various medicines should not be used.

I- When you give medicine, do you teach about the required doses of medicines and the methods to use antibiotics?

P- Yes, I tell them about the required doses of antibiotics but they do not take the required doses. They just take 1 to 2 dose of medicines and if necessary, then next day, they come to take medicines. Many people don’t take medicines after 1 to 2 doses. Very less people take the full doses of medicines. They also have problem of finance. They have love for their animals because their animals are expensive but they do not take the total doses of medicines. They also go for traditional healing of their domestic animals. It is also a conservative place.

I- Do these medicines work even with just 1 to 2 doses?

P- In 1 to 2 doses, these medicines reduce the symptoms but it does not completely solve their problems.

I- Those farmers who have low finance and low socio economic status, does the government give any subsidies to farmers with low socio economic status to motivate them? [9:48]

P- No, I have not heard anything about that. I refer those farmers to veterinary hospitals. In veterinary hospital, I don’t know how much services and what kind of services are given there in veterinary hospitals to the farmers. I am unaware about their services.

I- Antibiotics can also be used in bacterial infections. Like human beings, animals may also face bacterial, fungal, viral infections. In those conditions, what kind of medicines are given? Are the medicines for animals and human similar or different?

P- Yes, animals may also have bacterial, viral and fungal infections. Medicines for humans and animals are different. For antibiotic, streptomycin, procaine penicillin, etc., cortisone for viral infections etc. are used. In those infections, I refer the farmers to veterinary doctors in veterinary hospitals for better treatment.

I- How common are the fungal infections in animals?

P- Yes, various fungal infections are common in animals. People come to me with these problems like diseases in tail, infections in foot of animals etc. If I can handle, then I give medicine otherwise,

I- People are giving antibiotics to their animals. Do they have information about the antibiotics and other medicines used?

P- No, most of the people do not have enough information about antibiotics but they have mobile phone. So, they click the pictures of medicines which they are using and bring the pictures to us for medicines. People also go to border of India to buy medicines. For example: I have calcium of 5 liters with 1500 rupees. Other persons also sell calcium in 200 rupees. So people like to buy medicines with low price. People mainly focus on price rather than the quality of medicines. People also use unregistered medicines. Some people sell medicines which are not registered by Medicines Management Division. People who are selling agricultural products are also selling medicines which belong to unregistered medical practices. There are not much quality control and regulation mechanism. [12:38]

I- We are going in communities after sometimes. So, we want to find out whether they know about antibiotics or not. Do they have informations about antibiotics?

P- Some people have informations about antibiotics. About 1 in 10 people may have knowledge about antibiotics. You have to give them awareness and informations about antibiotics. People are slowly gaining informations about antibiotics. They should understand. I have given awareness to many people while doing the job of agro vet store. Now, you should also give informations to them to make them understand.

I- While buying antibiotics in humans, packaging is different for different antibiotics according to companies. Like, if we take photos of very common antibiotics and tell them that these medicines are used then can they understand about the medicines?

P- They may not have information. They may not understand about those medicines. They do not give attention towards the used medicines. They do not make any register or they do not keep any informations about the medicines. For example, in case of anti- rabies vaccine for dog, vaccine for hen, vaccine for cows whose insurance has been done, those vaccines are understood by the people. The people who do insurance to cows etc. keep record for vaccine used by cows. They get allowance from government. So, they keep record and understand about the insurance, vaccines and medicines. Other common people do not have much informations about medicines and antibiotics related to animals.

I- What are the differences between antibiotics used in hen, goat, buffalo, cow?

P- Antibiotics are usually similar which are used in hen, buffalo, goat, cow etc. but their doses are different.

I- In which animals, are more antibiotics used?

P- Before few years, there were more buffaloes and bulls. Now, there are lack of bulls. Tractor is mainly used in field instead of bulls for farming purpose. At present situation, animal farming is mainly focused in meat, egg and milk production. Poultry farming, cattle farming, fisheries, etc are popular these days for their products.

I- Are antibiotics also used in fish farming?

P- Yes, antibiotics are also used in fishes.

I- Are antibiotics also used in fish farming when fishes are diseasesd?

P- Yes, when fishes are diseased, antibiotics are used. Otherwise, antibiotics are not used.

I- Are antibiotics also used in poultry during any disease condition?

P- Yes, absolutely.

I- People say that if antibitoics are injected in hens, then they grow very fast. Is it true?

P- Antibiotics are only given during diseased condition.

I- Are there growth promoter medicines for hens?

P- Yes, there are many growth promoter medicines for cattle, hens, fishes etc. Some growth promoter medicines work for all the animals while some growth promoting medicines are only for hens. Some growth promoting medicines are only for fishes. We have to give those medicines to farmers by explaining about their importances to specific animals. These medicines have different doses for specific animals which should be explained to farmers. Some food supplements have 30 to 50 grams dose whose 1 packet should be mixed in 10 quintal grains.

I- Are there trainings available for farmers involved in agriculture and animal husbandry?

P- There are not much trainings available. Some trainings are organized once or twice in a year, and when trainings are organized, we just go to the program to take allowance. We just do some introduction and take little informations. There are no specific effective training programs which are organized regularly by government. We also have weakness that we do not go on training programs if there are no allowances.

I- Those common people who are not educated, if they are involved in agriculture, then, are they provided with trainings related to agriculture and animal husbandry?

P- There should be license of people to get training at present situation. There is a policy regarding licensing of agricultural practices. People should take license from DDA [Department of Drug Administration]. All the people do not get training of agriculture and animal husbandry. Government have specific quotas to provide training of agriculture and animal husbandry. Some people also take other’s license to get training on agriculture and animal husbandry practices.

I- Are there private clinics for veterinary?

P- Yes, there are private clinics which are being run by veterinary doctors.

I- Do veterinary doctors prescribe medicines?

P- Yes, veterinary doctors prescribe medicines. Doctors have opened their clinics. They should also take training and take the license. There are issues regarding the prescription of medicines by doctors which are being solved by the court. There are issues in law of Nepal with which, people have dissatisfaction. At present situation, doctors can’t open veterinary clinics without taking orientation and license from DDA [Department of Drug Administration]. This law of Nepal is not so fair because we had just taken the course of 35 days for JTA [Junior Technical Assistant] and we are running the clinic but the doctors who have studied veterinary for 5 years are not allowed to open the clinic and run them. These policies of Nepal government are contradictory. Many people who have not even studied 1 word are also running veterinary clinic by misusing the license of other people. People have done business of crores without having authority. Authorized organizations of government should check and measure such misconducts.

I- While prescribing medicines by doctor to farmers, they may also prescribe more medicines or wrong medicines.

P- Yes, mistakes may happen with everyone. Doses of medicines may be mistaken to prescribe for right quantity.

I- There are companies of drug production. Do doctors have relation with the production company of medicines?

P- Yes, there may be relation between doctors and production company of drugs. I don’t have much informations about their relation. Market representatives of production company of drugs also come to meet us and do advertisement about their products with us. We also check the quality of drugs and registration in DDA [Department of Drug Administration] and keep the products if it is applicable otherwise we do not keep it. Our doctors also come to visit us to check the medicines and sometimes consult us not to use some medicines which may be illegal or have low quality.

I- I would like to continue my previous question. In case of human as well, we go to doctors saying it is fever but in order to diagnose the infection whether it is bacterial or viral, we have to do various tests. In case of animals, do we conduct bacterial culture or test in practical setting or not before prescribing antibiotics?

P- In veterinary, 95% medicines are given by diagnosing the problems by looking at their symptoms, case history etc. We take little information about the case history of animals with the farmers. We observe the symptoms of cattle, their feeding behavior etc. before giving medicines. We do not even have pregnancy test.

I- How do you check the pregnancy of cattle?

P- We check the pregnancy of cattle by inserting hand after 5 to 7 months after looking at the size of stomach. It is not a good practice but we do not have other options. We do not have any test. We do not have X- ray service, ultrasound, pathology service etc. Diagnosis all depends on our prediction. Like one guy of village give 1 antibiotic, if that antibiotic does not work, then the animal is taken to market or =Taulihawa= then district hospital. It is very hard for the animal to take it to Kathmandu. It feels very sad because there is no investigation of disease in animals. It takes about 7 days to get the test result. When the test result comes, the animal may even die. I feel very sad about the death of animal without proper treatment.

I- When birds and animals get sick at home, who comes at the health center, whether male or female?

P- Today’s generation is not about male or female. Any gender come here to take medicine for their animal.

I- I am asking this question to know whether male or female take care of their animal so that when we go to community, it will be easier for us to get the information from family member.

P- Usually, parents, grandparents who stay at their home take care of their animals but to take the medicine from veterinary shop, young son or daughter of the home comes. Now-a-days, children are being educated. So, they are coming forward for these works.

I- When you give dose of medicines, do they understand about it and follow it regularly?

P- It will be difficult for a person to understand about the doses at one visit. I should not feel irritated about the situation. It is not his/her fault about not understanding the doses because s/he may not have studied. S/he may not have educational background. I should make him/her understand by explaining it continuously. It is my ethics to make him/her understand about the doses of medicines. For example, cypermethrin pyrethroid is a medicine which should be rubbed in the body of animals and prevent them from licking, and then the animal should be bathed. Such informations should be given continuously to prevent any complications or problems.

I- For example, if a person takes a medicine and finishes it partially then how are the remaining medicines disposed?

P- Sometimes, accidents may occur due to remaining medicines. I tell every people to put the remaining medicines safely, not in the access of children, in cool condition, avoiding sun and moist environment. If some medicines, antibiotics, vitamins, minerals are remained after using, then I suggest people to use those medicines by digging and burying them under the soil while planting trees of mangoes etc. I suggest people not to throw those medicines randomly otherwise, dogs in the street may eat those medicines which may have poisonous or negative effects in those dogs. For example: there is a medicine called diclovet, when diclovet is injected in any animal, if that cow dies after the injection of diclovet, if vulture eats the corpse of the cow injected with diclovet, then the vulture dies. It is also one of the reasons behind the extinction of vulture species. There are medicines with hazardous chemicals used in humans, but humans are burnt in Nepalese culture, so transmission of hazardous chemicals does not take place from human body after death. [32:00]

I- What are the behaviours of people about the safe utilization of medical products and their safe disposal?

P- People should be made aware about the safe utilization of medicines and antibiotics. People should be made aware about the negative consequences of haphazard use of antibiotics. People should be told that they should go to hospital and consult doctor before taking antibiotics. If we do careless in the safe utilization of antibiotics, then it may even take the life of a person. If we organize awareness programs to aware people about antibiotics, then I think people will understand about its importance and negative consequences.

I- Why are we not being able to reduce or eliminate antibiotic resistance? What are the main reasons behind the increase in antibiotic resistance?

P- For example; hen used to be of 2 kg to 2.5 kg after 60 to 75 days in past. But now, people want everything to be fast to increase their income. For that, people are using medicines and antibiotics randomly. People are giving hormonal medicines to hens to raise them faster which have consequently negative effects on ourselves. Chickens are raised by farmers, medical person gave those antibiotics to farmers and farmers used them in chicken, and their meat affected to local people of our community. Many antibiotics are increasing to be used in market like =ampicillin=, =ampicillin cloxacillin=, =ceftrizone=, =trazobactum=. These antibiotics are even giving to 3 grams to 5 grams which is also not working in patients.

I- Why are people using antibiotics so randomly?

P- Antibiotics are given due to disease condition as well. On the other hand, some people want to increase their economy so fast by injecting antibiotics to animals, poultry, cattle etc.

I- We have this trend in our society.

P- This trend is increasing all over the world. There is increasing trend of reckless use of antibiotics and their negative consequences.

I- This trend is mainly increased through the supply side like medical practitioners, doctors, pharmaceutical production companies, etc. for income generation. There is no fault of public in these activities. Can general public do any activities to reduce this antimicrobial resistance?

P- Antimicrobial resistance is mainly increased due to medical practitioners, doctors, businessmen etc. Training should be given to medical practitioners, municipality, ward office, general public about the safe utilization of antibiotics. We should be able to convince people about the negative consequences of haphazard use of antibiotics. Now, educated people have been aware about the safe utilization of antibiotics. They do not use random antibiotics without prescription. We should go from root level. We should aware rural people. But main thing lies in the economy. When a person goes to hospital, doctor will suggest about the pathology test, lab test, X- ray etc. A common rural person may not even have 500 rupees in pocket but when gone to hospital, the person requires at least around 4000 to 5000 rupees for all the common lab test and procedures. That’s where the main issue lies.

I- Anyway, thank you for your time and cooperation.

P- Thank you.

**Interview Transcript 2**

Notes taken after the interview, transcript not available

**Interview Transcript 3**

Interview details

| Participant ID | 3 |
| --- | --- |
| Occupation / role | Local pharmacist |
| Gender | M |
| Area | Urban |
| Service provider | Pharmacy |

Index:

I: Interviewer

P: Participant

I- “Namaste”

P- “Namaste”

I- What are the main problems which are brought by people in this pharmacy?

P- Mainly, children come to my pharmacy who are suffering from cough, pneumonia, diarrheal disease, cholera etc.

I- What is the ratio of children and adult clients?

P- More children come to me because I work with Pediatric specialist. Some adults also come to me.

I- When patients come, do they directly demand medicines or explain their problems to you?

P- People explain their health problems at first. Then, I give medicines according to their problems. If they have minor health problems like fever, headache, then they demand paracetamol by taking the name of the medicines.

I-  Which medicines do you use mostly?

P- I use paracetamol, antibiotics [cefixime, ciprofloxacin, cotrimoxazolle, amoxicillin].

I- In case of diarrhea, which medicine do you use?

P- In case of diarrhea, I use metronidazole, ORS [Oral Rehydration Solution], ciprofloxacin. In severe cases, I have saline.

I- Do sick people understand about antibiotics?

P- I don't give antibiotics to people in simple cases. If a patient has continuous fever for long period of time, then at that time, I may use antibiotics.

I- How do you explain people when you give medicine?

P- I give all the informations about medicines to the sick people. Like, when they bring children and if the children have common cold then, I tell them to use medicine for 3 days. 1 medicine is antibiotic. If the medicine does not work in 3 days, then another antibiotic will be added. We cannot give more antibiotics to children. If the situations don't get better, then another antibiotic will be added. That's how I do counselling to my patients. I give tropical syrup, multivitamins etc. to patients.

I- When you give antibiotics, how do you give them counselling?

P- If the patient comes to me after 3 days and the situation of the patient does not get better, then I add antibiotics [cefixime] to the patient. I give them antibiotics according to the age of the patient.

I- What is the normal dose of antibiotics that you give to patient?

P- The least dose of antibiotics is up to 3 days. Full dose of antibiotics can take 7, 10 to 15 days. If the condition of patient didn't get better, then another antibiotic can be added if the symptoms persist for long period of time. If there is fever and in 3 days, if there is no response, then I suggest them to do blood test, urine test.

I- When you explain about the doses of medicines, do they take the antibiotics according to your advice? or, they just take antibiotics for 1,2 days?

P- When they take liquid antibiotics, they have to take the medicines for 3 days compulsorily. Adults can take the medicine for 1,2 days and can add after 2 days due to lack of money.

I- In case of children, full doses of medicines are used but do adults also buy full doses of medicines?

P- No, adults don't buy full doses of medicines most of the time. They take medicines for 1,2 days and observe the response and come back to take the medicine again.

I- What are the differences between health seeking behaviors of people in case of adult health problems and children's health problems? What are the treatment procedures for health problems of children and adults?

P- I suggest people to do blood test and urine test after having antibiotic in both the cases of adults and children. Some children take medicines when they come to me. I ask them about the medicines which they took. I tell them to do blood test if the antibiotics are not working for 3 days. I tell them not to take risk for more than 3 days. In serious case, I tell adults to go to hospital. In serious cases, people don't come to me. They go immediately to hospital in case of emergency.

I- Where do the sick people go at first?

P- At first, sick people come to us and take medicines for few days. If the medicines don't work, then they go to hospital. In case of minor health problems, people come to us.

I- In which cases, antibiotics are mostly used?

P- Antibiotics are mostly used in case of fever for 3 days.

I- In which cases, antibiotics are mostly used in children?

P- Antibiotics are mostly used in children in case of fever, common cold.

I- In which cases, antibiotics are mostly used in adults?

P- Antibiotics are mostly used in adults in case of fever, common cold for more than 3 days. I do counselling to adults and children before giving antibiotics.

I- Do some patients come to your pharmacy directly by demanding the name of antibiotics?

P- Yes, some patients come to my pharmacy directly by demanding the name of antibiotics which have given better results for them. I give them the antibiotics according to their demand.

I- I am asking this question to you because next time, we have to go to community so we want to know whether people understand about antibiotics or not?

P- We tell people about the conditions when antibiotics are given.

I- Do people in this area understand about antibiotics?

P- Yes, people in this area understand about antibiotics. They have a concept that they should not use antibiotics. If no other medicine works, only in that case, antibiotics should be used. People in this area are well-educated so they have better knowledge and informations. [9:20]

I- Do people come to ask for simple antibiotics like metronidazole?

P- Yes, they come to ask for simple antibiotics like metronidazole. They also bring cover of antibiotics to demand for the medicines.

I- Who comes to pharmacy by taking the children? Is it mother or father?

P- Most of the time, mother brings the sick baby to medical store.

I- In case of adult, who come as guardian with the sick person?

P- Sick adults come themselves in medical store. In case of sick children, mother comes with sick baby.

I- Where are the other centers in this place where people can take antibiotics easily?

P- Another medical store is nearby. Another medical store is in =Bhabani Bhichhu= [Name of a place]. There are a lot of medical store.

I- Why do people come to medical stores instead of hospitals or health centers?

P- Due to the reasons of time and economic factors, people prefer to go to medical store at first instead of hospitals or health centers. When their health problems are solved by going to medical stores, they don't find it necessary to go to hospital and health centers.

I- Where is the health post of this place?

P- Health post is in =Keruwal= village.

I- If we have to ask about antibiotics to the community people in this area, then do they understand about the differences between general medicines like painkillers, paracetamol etc. and antibiotics?

P- Educated people understand about the differences between general medicines and antibiotics. Uneducated people may not understand but maximum people in this area are educated. So, most of them understand the differences between general medicines and antibiotics.

I- People may know about antibiotics. Do they know about anti-fungal, anti-protozoal medicine?

P- They may not know about the differences between antibiotic and anti-fungal medicines. We give them medicines. Educated people can read the label, so they can understand but uneducated people may not know about anti-fungal, anti-helminthic medicines. They may understand the medicine but they may not know the name of the medicine.

I- Medicines have different brand name and generic name. Different medicines of different company have different packaging. Are there availability of different brand or company of same medicine in this area?

P- Yes, there are various brands of medicine. Color and cover of the medicines differ according to the company. If customer demand any specific medicine, then we should bring the medicine of same brand, same color etc.

I- When we go to community, if we bring antibiotic and show it to community people, then they may not understand even by looking at the cover of medicine, right?

P- Yes, people may not understand because there are various brands and types of antibiotics available in the market.

I- How can we explain antibiotics to community people to make them understand?

P- You can tell them about the antibiotics and say that it is the antibiotics of different brand. You can tell people that various types of antibiotics are available.

I- Are there any specific local names for antibiotics in the community?

P- No, there is no specific name for antibiotics. Educated people can understand while uneducated people may not understand.

I- Do you give advice to people and tell them directions about the use of full doses of medicines to people?

P- Yes, I always give directions for the proper use of full doses of antibiotics.

I- People may not have taken the full doses of medicines even when you give the full doses of medicine. Do people regularly follow your advice?

P- Generally, we give them medicines for 3 days. If people come here with response, then they take the doses given by me. Some people may go to hospital by not taking the full doses of antibiotics.

I- Sometimes, medicines are left over after being used. How do people use those remaining medicines?

P- I give suggestions to people not to use the medicine from the part of expiry date. While taking the left over medicine, we have to look at the date of medicine and we should not consume it after expiry date. I tell this to my customers.

I-  I wanted to ask you that, when people take medicines for 5 days, eat it for 3 days and then, what do they do with the remaining medicines?

P- They come to ask me before eating the remaining medicines when kept for a long time. If they bring me back the remaining medicines, I keep it back. I also tell them to take the medicine if it has date remaining. I don't give suggestion to use antibiotics even if the date is remaining because there are various conditions which should be considered while using antibiotics.

[18:42]

I- How much knowledge about antibiotics do people have in this community?

P- Yes, most of the people have knowledge about antibiotics in this area because most of the people are educated in this place.

I- Do people share their medicines with neighborhood? Do they take medicines from FCHVs [Female Community Health Volunteers]?

P- People take suggestions from FCHVs [Female Community Health Volunteers] but they don't usually share medicines with neighborhood.

I- Do people in this area have informations about antibiotic resistance? Do people know that misuse of antibiotics can lead to antibiotic resistance?

P- Educated people know that misuse of antibiotics can lead to antibiotic resistance.

I- As a health professional, what do you think is the reason behind increasing antibiotic resistance? Why are we not being able to reduce antibiotic resistance?

P- People are also very careless. People go to one health professional and take medicine and in next 1,2 days, if s/he doesn't feel good, then s/he changes the doctor very quickly and changes the medicine very fast according to health workers.

I- Have you find people not following your advice to take the full doses of medicine?

P- Some people who have already taken medicine from other place tell me to add the same medicine. Other people take my advice when I tell them to take full doses of medicine.

I-  Do they tell you to add antibiotics for them?

P- No, I communicate with them and find out which antibiotics are they using and I give them medicines according to their medicines used in past.

I- Okay. Thank you so much for your time.

P- Thank you.

**Interview Transcript 4**

Notes takes after the interview, transcript not available.

**Interview Transcript 5**

Interview details

| Participant ID | 5 |
| --- | --- |
| Occupation / role | Qualified doctor from private clinic |
| Gender | M |
| Area | Urban |
| Service provider | Private clinic |

**Index:**

**I: Interviewer**

**P: Participant**

I- “Namaste! “

P-“Namaste ma’am!”

I- What are the major health problems of people with which they visit this clinic?

P- The major health problems of people depends on season. Before few months, people were coming to this clinic with fever, cough etc. during the time of COVID-19. Now-a-days, may be due to vaccination, people with fever, cough and symptoms of COVID-19 have been reduced. There are various regular follow up cases with diabetes, hypertension etc. COPD [Chronic Obstructive Pulmonary Diseases] is high in old people. Fever is also a common problem in people of this area. Acid peptic disease, gastro-intestinal problems are also very common in people. Some people come with non-communicable diseases like hypertension, etc.

I- What is the first contact point of people before coming to this clinic?

P- People take antibiotics at first and if antibiotics do not work, then they come here. There is a medical in every 2 villages. AHW [Auxiliary Health Workers] are present in every medical. People have fever as a very common problem. The mostly used and misused antibiotic is cefixime here. There are two factors like money and distance. To save these factors, people first visit the local medical store and if it didn’t work there, then only, they come to our clinic. AHW [Auxiliary Health Workers] in every medical store give 3 doses [3 tablets] of cifixime for 3 days. Some AHW who have the concept of dual antibiotic therapy gives cifixime and azithromycin. They have decold [anti-allergic and paracetamol], cifixime and aciloc. If a person suffering from fever goes to medical store, then there, AHW [Auxiliary Health Workers] prescribe them cifixime and azithromycin. If people with fever go to health center, then there is cotrimoxazole and amoxicillin. Cifixime is not available in government health center. So, the mostly used drugs in government health center are cotrimoxazole and amoxicillin. In last 2 years, I am analyzing the report of blood test. I have the record. I have done about 40 to 50 blood cultures in last 1 year and I have found cifixime resistance in 99.9% of the blood culture reports. Asian Diagnostic Lab is our reference laboratory. We use that lab and there are all our records that 99.9% of the tested blood is cifixime resistant.

I- As you said that, cifixime is a common antibiotic. Is it common in all these areas of Kapilbastu district or different medicines are also used in these areas?

P- Local AHWs [Auxiliary Health Workers] in this area have limited knowledge which is limited to cifixime and megapen in case of wounds, folliculitis. Some AHW [Auxiliary Health Workers] use amoxiclave but these AHWs [Auxiliary Health Workers] are limited to these medicines because they have limited knowledge and economic limits because cifixime is the cheapest antibiotic available in the market at present condition. Other antibiotics like Cefuroxime etc takes 40 to 50 rupees per tablet which is very expensive. Amoxiclav costs 30 rupees per tablet. AHWs [Auxiliary Health Workers] have to treat their patients in low cost around 100 rupees in this area. So, the most commonly used, misused and resistant antibiotic in this area is cifixime. [4:20]

I- As you said that, they give doses for 3 days at first. When they come to you, do patients take all the doses of medicines or they leave the medicine after 1,2 days and come to you?

P- It depends on the nature of illness. If it is the case of URTI [Upper Respiratory Tract Illness] or simple viral fever, the symptoms will be resolved after 1,2 days. Symptoms of some viral illness resolves in 5,6 days so the patient think that the AHWs [Auxiliary Health Workers] is a very good doctor and s/he stays at home and refers other people to the same health workers. But in case of other bacterial infections or atypical fever due to various organisms, bacteria like salmonella, leptospirosis, brucellosis, E. coli [Escherichia coli] or organisms which does not respond to antibiotics, these kind of fever does not resolve. Those patients suffering from such illness come to us after 7 to 10 days after wondering to different medical and health centers. Then, according to history, we observe the symptoms, fever etc. Fever duration is also the associated symptom of any disease. Then we have a concept that it is an infectious fever from 7 days, then I counsel the patients to do blood culture in most of the fever cases. There are two reasons behind it. One reason is that we do blood culture within less period of time. It takes 3 days to do blood culture. In this area, blood culture is not done in all the health centers. In hospital, it is easy to do blood culture by admitting the patients to hospital. Sometimes, I suggest CBC [Complete blood count], CRP [Creatinine. C-reactive Protein]. In case of UTI [Urinary Tract Infection], I prefer urinary test. Therefore, I do not recommend patients antibiotics randomly. If patients do not have money, then sometimes I have to recommend empirical antibiotics but cifixime never comes to my choice except pregnancy. During pregnancy, I give cifixime in UTI otherwise I don’t trust cifixime because in every blood culture that I have done, every report is cifixime resistant. Amoxiclav have the highest susceptibility among other simple antibiotics at present condition. Amoxicillin is also found to be resistant in reports of blood tests. If you interpret the report, then you will find out that most susceptible antibiotics are amikacin in injection, meropenem, linezolid, amoxiclav, gentamicin etc. Cifixime is the highly resistant antibiotic in this area.

I- Do patients understand that anti-cold tablets like decold, paracetamol are different from antibiotics?

P- Yes, many people understand about the antibiotics. People have good knowledge about cifixime. They tell me that they have taken antibiotics when asked to people. Some people show me tablets and say that they have eaten antibiotic tablet. They distinguish medicines with their shape and size. They can also distinguish the medicines of gastritis. Pantoprazole costs 8 rupees per tablet and aciloc costs 1 rupees per tablet. So, they know aciloc, flexon, paracetamol etc. So, they can distinguish antibiotics from other medicines.

I- Packaging are different due to brand name and generic name. So, is it difficult for people to distinguish between antibiotics due to brand name. I am asking this because while doing survey, we have to go to survey and ask people about whether they have used antibiotics or not. Are medicines of same brand used more in this area? Or, some medicines come to market with same generic name and different brand name. How common are the medicines with same generic name and different brand name in this area?

P- In Nepal, brand name is used widely. The concept of using generic names are not popular till now. Few educated people understand about the cifixime etc. Some people understand about aerosafe, acrosafe. They understand that these medicines are cifixime. Illiterate people do not understand about antibiotics but educated people easily understand about antibiotics.

I- Uneducated people can also understand about antibiotics by looking at their packets. [9:52] …[interrupted]

P- There are many antibiotics available in Nepal. There are around 100 to 200 pharmaceutical companies in Nepal. Among them, medicines of 25 to 30 pharmaceutical companies are available. People will not know about all the packets of antibiotics but some people understand and can distinguish between antibiotics and other common medicines. [10:38]

I- Anti-microbial drugs consist of various drugs like anti-fungal drugs, anti-parasitic drugs, anti-helminthic durgs etc. Are anti-fungal drugs, anti-parasitic drugs, anti-helminthic durgs etc. used in this area?

P- Fluconazole are anti-fungal drugs. We don’t use fluconazole. Fluconazole is famous just like cifixime. AHWs have weakly understood about the fluconazole. Fluconazole are not used in the dose of 1 week. If it is a proper fungal infection, then the doses of 1 week is not effective. We have not even studied the dose of 1 week of fluconazole. 1 week of fluconazole is not effective. Fluconazole is used OD [Once Daily] [5 to 10 mg per kg]. So, fluconazole is used in such way. Another problem is in fungi infection, they use the medicines of eczema like Derma-KT, sonaderm etc. When these medicines are used in rashes, it has negative effects on skin. So, it has high financial impacts on people. If people come to hospital at first approach, then effective medicines like itraconazole etc. can be used to solve the fungal infections. People go to pharmacy and ask for medicine of rashes with 50 rupees. It is not also the fault of pharmacist because a person may come with 50 rupees and ask for the anti- fungal drugs when anti- fungal drugs are not found in 50 rupees. Fluconazole is only a drug which comes at 50 rupees. Fluconazole is a steroid so; it is effective only for short period of time. They spend 1,2 months thinking the problem will be solved. Then only, they come to visit us saying they have gone everywhere to solve the rashes but the rashes are not gone and then they request us to treat the rashes. In that situation, we have to ask their financial aspects because anti-fungal medicines are very expensive. It costs 45 rupees per tablet and we have to use it for 1 month.

I- We can diagnose people about bacterial infection by asking their symptoms like fever, cough etc. But for fungal infection, how can we make them understand?

P- People easily understand the word rashes [daadh]. They do not understand the word ‘fungus’.

I- What are the symptoms of fungal infections?

P- The symptoms of fungal infections are rashes [taniea], eczema, scabies. People generally do not understand the fungal infection.

I- That means, we have to tell people the meaning of fungal infection as itching disease.

P- Yes, we can ask them whether they have itching disease or not with round structures on the surface of skin. If the rashes are all around the body, then the problem is scabies. If the rashes are on the foot from long period of time, then it is eczema. We can make them understand like this. If rashes are on the hands, legs, work on soil, the person is mechanic, rashes look dirty and rough then the problem is eczema. If the rashes are all over the body, in between fingers and it itches a lot at night, then the problem is scabies. If rashes are in the moist area, with round structure which is red in color at first, then it is taenia.

I- In case of fever, cefixime is given in many pharmacies and clinics as you said. Do people demand antibiotics themselves? People go to pharmacy to take medicine but pharmacists give them antibiotics. Do they [community people] know about that?

P- Maximum pharmacists give antibiotics when people come to them with fever. Fever can be viral for 1,2 days but they will prescribe antibiotics [cefixime] because if pharmacist gives paracetamol, aciloc, then s/he will not get much profit but when they give cefixime, then they can earn some profit. In hospital, we take charge at first, so it will be okay even if we give them medicine of 10 rupees. We tell them to take the medicine and observe the result at first. If simple medicine doesn’t work, then we will go for investigation and prescribe antibiotics.

I- Anti-fungal disease is related to itching problem. In case of diarrhea, which antibiotics are used?

P- Ciprofloxacin and metronidazole are used in this area. ORS [Oral Rehydration Solution] is also given for diarrhea. Cefixime is also used in such case. Cefixime has been used in all the conditions. That’s why, people are resistant to cefixime. [16:00] One negative aspect is that metronidazole is given by pharmacist when a child below 5 years of age suffers from diarrhea which is very harmful for children. Metronidazole should not be used in children. Children do not usually suffer from diarrhea due to parasitic antamoeba histolytica. Most of the time, children have viral diarrhea. [17:13] We should only give zinc, ORS [Oral Rehydration Solution] and a probiotic bifilac to children, then the diarrhea will be solved but they [medical practitioner] give antibiotic.

I- What are the differences between first contact point when a child gets sick and when an adult gets sick? In which place is a child taken, when s/he gets sick? Is s/he also taken to pharmacy like an adult?

P- People generally go to local medical practitioner who are running medical store. They go to same medical store for both adults as well as children in any of such problems.

I- In case of severe problems, or if the problem is not reduced for long time, then where do they go?

P- In case of such problems, then they come to visit us or they go to district hospital. If someone gets faint or a lady has pelvic bleeding, then s/he doesn’t go to medical store, they go to hospital because they feel like medical can’t solve their problem. If a person gets wound at head and the person is vomiting, then they rush to the hospital.

I- When you give antibiotic after doing blood test, culture, do patients follow your advice? Do patients follow your advice and take medicines regularly according to the required dose?

P- We look after frustrated people. When people come to us, they have already become frustrated. We have follow up cases of 50% patients of chronic diseases. Most of the people who come here have already been frustrated. When s/he gets diagnosed, I convince them that they have this germ in their body and they have to take these medicines after diagnosing from different tests, then they get convinced and they follow my advice and they become sure that their problems will be solved.

I- The practice of people is like, for example, required dose is for 7 days. People start to take medicine and after taking medicine for 2,3 days, they feel relief and then they leave the medicines…[interrupted]

P- Yes, there are people with such habits but we should do counselling to patients and tell them that they should take the medicines to the required dose. My personal habit is like, I give phone number to patients and take their numbers and do follow up on call as well. We have to do continuous counselling that they should take the medicines till the required days and doses otherwise, the germs will not die and the same medicines will not work if used again. So, we tell them to take the medicine to full dose.

I- What are the differences between trends of using anti-microbial drugs and antibiotic drugs between children and adults?

P- In medical stores, the trend of using anti-microbial drugs and antibiotic drugs between children and adults are same. Cefixime is most commonly given to both children and adults. The only difference is children are provided with liquid antibiotic while adults are provided with solid antibiotic.

I- There are people in our community with different religions and ethnicity like Brahmin, Chettri, Tharu, Hindu, Muslim etc. Are there differences in health and medical practices of people in terms of use of anti-microbial medicines?

P- People also have religious belief. They also go for traditional healing along with medical practices. There are not much differences in terms of religions and ethnicity but there are many differences in terms of education. For example: in ward no. 4, people are highly educated. There, people consult with doctors before using antibiotics. They do not carry out treatment activities without investigation. Practice of people depends on education and the environment but it does not depend on religion and ethnicity.

I- Some people may buy more medicines than necessary. Suppose, you gave medicine for 57 days, but s/he took the medicine for 5 days and kept the left over medicines and reused them. Or, how do they dispose the left over medicines?

P- Some people may keep the remaining medicines and re-use them after sometimes. My village is nearby. Some people come to my home to ask if the date of medicine is expired or not, or if the medicines can be reused or not?

I- How do people dispose the remaining medicines?

P- People may throw left-over medicines anywhere. They may throw the left over medicines at rubbish, tap, canal etc.

I- As a health worker, you said it earlier that there is cefixime resistance. What are the main reasons behind the antibiotic resistance? Why are we not being able to end the antibiotic resistance?

P- There is availability of antibiotics everywhere. Anyone is giving and prescribing antibiotic. In COVID-19, ceftriazone was used so widely in this area that this medicine became scarce. Ceftriazone costs 35 rupees but during COVID-19, ceftriaxone was not found even at 100 rupees.

I- Antibiotics during the time of COVID-19?

P- Yes. So there are no proper policies about who can use the antibiotics and who cannot. Antibiotics are available everywhere and anyone is using antibiotic randomly without standard treatment protocol.

I- From the side of community, are there any factors due to which antibiotic resistance is increasing?

P- People are not educated and aware about antibiotics and antibiotic resistance. People do not even know that they are using antibiotics randomly. Medical personnel are using antibiotics without standard treatment protocol. Nepal is not a highly educated country. In many places, people are not clear about the concept of medicines and antibiotics. It is not their fault.

I- Are there people who demand antibiotics themselves?

P- There are some people who demand antibiotics themselves. For example: I have a patient of COPD [Chronic Obstructive Pulmonary Diseases] who ask me to give azithromycin because it becomes easy for him when he uses azithromycin during COPD [Chronic Obstructive Pulmonary Diseases] attack.

I-Is it common that people demand antibiotics themselves?

P-No, we don’t give antibiotic randomly to patients even if they demand it. We give counselling to them and tell them to take antibiotics and medicines according to the required doses and advice.

I- Thank you for your time and cooperation.

P- Thank you.

**Interview Transcript 6**

Interview details

| Participant ID | 6, 7 |
| --- | --- |
| Occupation / role | Health post in-charge (health assistant) |
| Gender | M |
| Area | Urban |
| Service provider | Health post |

Index:

I: Interviewer

P1: Participant 1

P2: Participant 2

I- “Namaste”

P1-“Namaste”

P2-“Namaste”

I- I- Can you please tell me the name of your health post?

P1- The name of my health post is =Neklewa= health post. It lies in the ward no. 9. It lies inside =Kapilbastu=. We have 12 wards in =Kapilbastu=.

P2- The name of my health post is =Sauraha= health post. It lies in ward no. 12. It also lies inside =Kapilbastu=.

I- How long have you been working here?

P1- I have worked here since last 6.5 years. I have come to Kapilbastu 2 years ago.

I- Do you know =Shambhu= brother?

P1- Yes, I have known =Shambhu= brother as he is the member of our ward.

I- How long have you been working here? [asking to second participants]

P2- I have been working since last 7 years. I have come to =Kapilbastu= 5 years ago.

I- What type of problems do people mainly come with in health post?

P2- People come health posts with the health problem of diarrhoea, dysentry, scabies, fungal infections etc.

P1- Like he said, there are fungal infections. When I was in duty in hilly region for 4 years, in my experience, there were very few problems of skin. Only 200 to 300 tip of whitfield ointment, cotrimazole from 1 municipality of hill. 200 to 300 tip of whitfield ointment, cotrimazole was enough for 1 years and we used to get some surplus ointment from it. But last year, in Shrawan of 2077, 100 tips of cotrimazole and 100 tips of whitfield ointment [mixture of salicylic acid and benzioc acid] was finished in 35 to 40 days. I find a lot of skin problems here.

I- Which is the place where people go at first, in this area?

[2:12]

P2- At first, people go to private medical store. If their problems are not solved there, then they come to us. Many people are educated and aware. They have knowledge, so they come to us.

I- When people go to private medical store, they use medicines by themselves.

P2- Yes

I- What type of medicines do they buy and use themselves in private clinics?

P2- Specially in the southern belt of terai, people use antibiotics at first like monosafe, cefixime, amoxicillin etc. They buy and use these antibiotics themselves.

I- That means they buy and eat antibiotics themselves.

P2- Yes

I- Do people go and ask for antibiotics themselves or medical personnel suggest them [people] to use antibiotics?

P2- Are you asking in case of private medical store or government health center?

I- I am asking in case of private clinic at first. [3:30]

P- In case of private medical store, people also demand antibiotics by themselves. Medical personnel also usually give antibiotics to solve the health problems faster.
I-In your case, do people go and ask for antibiotics themselves or medical personnel suggest them [people] to use antibiotics? [ asking to first participant] [4:09]

P1- I would like to tell you both the case of =hill= and =terai=. In case of hill =[Pyuthan]=, People do not know about the term "antibiotic" but they used to say that he is suffering from typhoid [bailo] and they wanted cipro [ciprofloxacin]. [4:48]       In =Terai=, in place where I live, there are two communities which are hilly community in ward no. 6 and madhesi people in ward no.1. Very few hilly people come to health center. Few women of hill come to us. They used to come to our health center before but in the past, we did not have medicine and we had to return them without medicines and maybe they did not understand our problem so they did not come to us after that. If they come to us, then they say they have this problem so,this medicine should be given to them.

I- Do they [hilly people] ask you to give certain medicine?

P1 - They say like, they have diarrhoea, so they want 'metro'[metronidazole]. We try to investigate their problems but they directly say that they want certain medicine so they don't let us do the investigation sometimes. They may also find out informations about medicines from social media, youtube etc. In some cases, like, in our CB- IMNCI [Community Based Integrated Management of Childhood Illness] protocol, it is said that we just have to give zinc. So, we just give zinc and 2 packets of ORS [Oral Rehydration Solution] but when we go out, people say that doctor did not give any medicine even when health center have that medicine. Protocol also include that we should not give cetamol to children with fever but they send us cetamol. I don't know why they send us cetamol. We give cetamol. We also have enough metronidazole. We work according to protocol. So, metronidazole has also been expired. Cetamol has not been expired because we give cetamol to people during fever.

I- Do terai people clearly state about the medicine they want like cetamol?

P1- They say that they have certain problem like common cold, but they do not ask to give certain medicine like cetamol. But matured, educated people of terai also know about all the problems and medicines.

I- What about your health center? [Asking to second participant]

P2- In our health center, they directly ask by taking the name of medicine like metronidazole, aciloc etc. We give them advice but most of the people directly ask to give them certain medicines.

I- In which conditions, do people come to you? You said that they have already gone to medical store and they have already taken antibiotics by themselves. Health worker may give antibiotics but the patient may not know that the given medicine is antibiotic.

P1- People don't know that specific medicine is antibiotic. Like few days ago, I went to village to give medicine of lymphatic filariasis, I gave people the medicine of lymphatic filariasis, I may not have told every information about the medicine of lymphatic filariasis, I may have forgot to tell them all the information about the medicine of lymphatic filariasis. One person suffered from fever. That day, he took the medicine in the evening. Next day, he went to medical store due to fever and took medicine like flexon, cefixime etc. Medical personnel also give medicine for their business purpose.

P2: While comparing government and private health center, I find out that private medical store gives metronidazole, ofloxacin but they did not give zinc. In that case, if the diarrhoea is viral, then diarhhoea does not get solved, then when people come to our health post, we give them zinc and other medicines in systematic way and their problem gets solved. They also praise us in such conditions. It is a small example.

I- That means, educated people understand about medicines like antibiotics. Do uneducated people also have idea and information about the medicines like antibiotics?

P1- I think uneducated people cannot tell about medicines like antibiotics. Educated people can give a lot of informations about medicines but when an uneducated person becomes sick and come to health center, it becomes difficult for them to understand letters written using pen instead of markers in the bottle of medicine and they ask us 3,4 times about the medicine. They may not remember the medicines and their doses. They ask us frequently about the medicines. In our government health center, we don't use antibiotics in huge quantity.

I- Do you give antibiotics if patients demand for it?

P1- No, patients cannot demand antibiotics by themselves. We also don't give antibiotics randomly. Sometimes, if they come to us to take medicine, we should also give them medicine otherwise they do not trust us. One problem is that, some people come to take medicine by keeping patients at home like buying food from shop. For example; there is an old mother who come to us to buy medicine saying that she has small child at home who is suffering from various health problems. In such cases, it becomes very difficult for us to decide whether we should give medicine or not. They may complain that we did not give medicine. Some people may quarrel with us for not giving their demanded medicine.

I- As you said earlier that, people demand metronidazole by themselves. Likewise, do people demand antibiotics and when they demand, what do you do in such case?

P1: There are two types of people who come to demand medicine. Some people demand antibiotics because of their actual problem like diarrhea. Some people demand antibiotics or other medicine because it will be difficult for them to come to take medicine for the next time.

I- How would the person know that certain medicine can be used in certain health problems when s/he takes unnecessary medicines at home?

P1: The person knows that the medicine is of diarrhea and s/he demands that medicine so that s/he can use the medicine during emergency.

I- Do you face similar case in your health center? [Asking to second participant]

P2- Yes, it is similar in our case as well. People ask for amoxicillin, cetamol and other medicines by themselves. At that time, we ask them their health problems and reasons behind the demand of specific medicines.

I- Which antibiotics are most widely used in health post?

P1- Amoxicillin is the most widely used antibiotic in our health center. Sometimes, scarcity of amoxicillin occurs. Like, we faced scarcity of amoxicillin of 5-6 mg capsules in the year before last year. This year, we have adequate amoxicillin which is enough for 3,4 month but we do not use amoxicillin unnecessarily.

P2- Amoxicillin, ciprofloxacin etc are most commonly used antibiotics.

I- How much metronidazole are used?

P1- When our country was declared open defecation free zone, then the consumption of metronidazole has been reduced. Metronidazole has been kept in stock since last year in our health center. The use of metronidazole has been reduced. But people keep on demanding metronidazole and this medicine in bottle is also being used for children. Even if I had taken training and learned to not to give the metronidazole for child, if a child has faced diarrhoea for 10 times, then I also give zinc and metronidazole to solve the problem faster otherwise I don't recommend metronidazole for children.

I- Antibiotic is used for bacterial infections. Besides antibiotics, what are the other anti- microbial drugs like anti protozoal, anti- fungal, anti- parasitic drugs which are mostly used in health centers?

P1- For anti-protozoal drugs, sometimes tinidazole is used. Metronidazole is the most common medicine.

I- As you said earlier that patients themselves demand antibiotics like amoxicillin and metronidazole. When patients demand these medicines, how much dose of antibiotics do they take and how do they maintain the dose of antibiotics?

P1- You have asked a serious question. We know that the course of antibiotics should be used for at least 1 week but  even when we know this information, we have to do provisional diagnosis of patients. We cannot do the actual diagnosis. While doing provisional diagnosis, we give amoxicillin in case of tonsilitis, sinusitis, bronchitis etc. We can give the dose of 1 week when the medicine is enough. Otherwise, we give them the dose of 3 days.

I- Do they come for follow up?

P1- Some people don't come for follow up if they feel better in 2 days. Some people go to medical store if they don't feel better after using medicine for 2 days. That's why, the cases of follow up is very low.
I- In your case? [16:31]

P2- People come and we give them the dose for 3 days. We also have to look at the stock. We cannot give medicine dose for 7 days because we have to give medicines for other people as well.

I- That means, it is a type of obligation because there are lack of medicines to give full dose to all the people.

P2- Yes

I- As you said earlier, that mother of patient comes to demand medicine without bringing patient.  In most of the cases, who comes to take medicine? Do patients come to take medicine or family member comes to take the medicine?

P1- In 50% of the cases, patients come to take the medicine but sometimes, like I said earlier that, some people come to take medicine by saying that they have patient at home. According to our rule, we cannot give medicine to the family member who have patient at home because they behave as buying food from shop.

P2- This system has changed a little bit. Now-a-days, sick people come at health center themselves.

I- Does patient come alone or s/he takes friend with him/her while visiting health center?

P2- Patient also brings his/her friend while visiting health center.

I- Like, in a home, if a son gets sick, then which parent come to the health center, most of the time with his/her son?

P1- Mother comes to health center with her son most of the time because fathers are mostly engaged in job, some fathers may have gone to abroad so they may stay busy most of the time. In some cases, small children come to health center alone. In such condition, it becomes difficult for us because the child may not understand about the doses of medicine. So, a parent should visit health center with his/her child. [18:15]

I- In case of fever and diarrhoea, most of the people go to medical store at first as you said earlier. Is there any difference in child and adult?

P2- Most of the educated people with high economic status prefer to go medical store in any condition. People with low economic status come to us.

I- Sometimes, adults go to medical store and buy some medicines in case of tonsils and if the medicine work, they don't go to hospital. But in case of children, people don't want to take risk so they rush to hospital. Is there any difference in this trend?

P2- Yes, there is difference in this trend. However, the group of people who come to our health center are different from the group of people who go to medical store for medicines. There are separate group of people who visit us and there are separate group of people who visit medical store. When I was working in other areas as well, according to my experience, the group visiting health center and medical store are different. Sometimes, people who don't get medicine in medical store come to us and sometimes, people who don't get medicine from us go to medical store.

I- Which are the places in community where people can buy and have medicines easily?

P1- Besides health post and medical store, FCHVs [Female Community Health Volunteers] give some simple medicines like cetamol to community people if they get fever and other simple health problems.

I- As you said earlier that people sometimes take extra medicines from health centers, if his/her neighbour get sick, then s/he may share his/her medicine to neighbourhood, are these practices prevalent in society?

P1- Yes, we can find few people who share their medicines with neighboring people during some health problems like fever, diarrhea etc.

I- Do people also share antibiotics with each other as they share other medicines like cetamol?

P- No, people do not share antibiotics with each other in most of the cases. People may share metronidazole with each other in case of diarrhea.

I- that means, metronidazole is the commonly misused antibiotics.

P1- Yes, people share and use metronidazole by sharing with each other.

I- Do people go to different health care centers on the basis of severity of health problems?

P1- Yes, people go to different health care centers on the basis of severity of health problems.

P1- In case of common cold, fever, gastritis, diarrhea, scabies, fungal infection, people come to our health center, otherwise in case of complex health problems, people don't come to us. They visit hospital during complex health problems. In our health centers, 20-50 children come to us monthly while observing the protocol of CB-IMNCI [Community Based Integrated Management of Childhood and Neonatal Illness], we just find 4 to 5 cases of pneumonia in last fiscal year 2077/78. Obviously, there are children with pneumonia in the community but they don't come to us. They go to hospital because of the severity of the health problem.

I- Do people have more beliefs on medical store than government health center in this area?

P1- Yes, people have more belief on medical store than government health center not only in this area, but also in other parts of the country but we can find a lot of people in government hospital as well. There are better health facilities in government health centers and hospitals as well. We have enough medicines in this month according to the citizen charter.

I- How much medicines do you have which are listed in the citizen charter?

P1- We have 36 types of medicines which are listed in the citizen charter.

I- Among these medicines, how many antibiotics, anti- fungal, anti-protozoal medicines are found?

P1- We have to count these medicines. We have around 3 to 4 types of antibiotics. We have 2 types of topical fungal medicines. We have 1 anti-parasitic medicine [albendazole].

I- Do people ask for the medicine of ascariasis themselves?

P1: Yes, people ask for the medicine of ascariasis themselves in our health center.

I- You don't use antibiotics much according to your protocol. But while using antibiotics, how do you explain patients about antibiotics? Do you use generic name or brand name of antibiotics?

P2- We write the generic name of antibiotic. People usually do not understand the name of antibiotics.

I- I am asking this question because we are going to do survey in community. People don't usually understand the name of antibiotics. Some people may not even understand the meaning of antibiotics. That's why, if we take a common antibiotics or its photo to community and ask people whether they have taken these medicines or not, then will they be able to understand?

P1- Yes, they will understand in such way. 
P1- We have 1 problem in our clients that if we say them not to use the antibiotics in simple health problems then they directly go to medical store and buy antibiotics from there. They become unsatisfied with us for not giving antibiotics to them. They easily understand the packet of antibiotics because most of the people have used those antibiotics.

I- Another problem is that; different packets of antibiotics are found according to different brand names. In your health center, do you get antibiotics with different packets? Or, do you always get antibiotics with same packets?

P2- No, packets of antibiotics changes according to the company. We get antibiotics with different color. Color of the antibiotics also change according to the company.

  In past, we used atenolol as anti-hypertensive drug. In present condition, we use amlod as anti-hypertensive drug. Some elderly people have returned the amlod saying it is different from atenolol. They said that they had used different medicine in past. They can't study so they depend on color to find out specific medicine.

I- You have worked with both the community as madhesi [terai] and hilly community. Have you seen any difference in the health seeking behavior of madhesi [terai] and hilly people?

P1- Educated hilly people have good health seeking behaviour of people. Some of the hilly people also have low health seeking behaviour in comparison to terai people but most of the hilly people are educated and have good health seeking practices in comparison to terai people.

P2- While talking about antibiotics, terai people use antibiotics randomly in comparison to hilly people because there is border nearby so availability and use of antibiotics is high in random manner in terai people.

I- Are these practising medical workers all trained health workers?

P1- Most of the practising medical workers are all trained health workers.

I- Like in the border area, there are availability of quack doctor as well. These doctors give injection which contains steroid so it works little faster so people have high trust on them. While working in Bardia, I had seen such doctors very commonly.

P1- Here, we can find a lot of such quack doctors and people have high belief on them.

In =Budhavumi=, there is one such doctor. He took diclofenac injection and polivine and asked to inject polivine directly in the vein. I said polivine should not be used directly. It has negative effects as well but he said, he had used these injections 3, 4 times. So, not to worry about that. Then I injected polivine.

P2- These quack doctors have been reduced these days. People don't go to these doctors now-a-days.

I- Are these quack [fraud] doctors available in these area now-a-days?

P1- In upper region, there are not much fraud doctors but in lower region [border areas], these doctors can be seen. According to rule, these fake doctors cannot run medical shop but in reality, some fake medical doctors can be found in border areas.

I- Can community people in this area differentiate between antibiotics and other simple medicines like cetamol, medicine of gastritis, allergy etc.?

P1- If people are educated and aware, then they have awareness about different medicines regardless of their society. There are various misconceptions and superstitions in our own community.

I- It means, general people do not understand about the differences between antibiotics and other common medicines.

P1- Yes

I-  When people take antibiotics, sometimes, medicines are left over after use. How do they dispose the left over medicines? Do they check the date of left over medicine and use it again?
P1- People have not come to me by asking the date of remaining medicine. They have not asked me whether they can reuse the medicine or not. So, I don't have much idea about it.

I- Do they have knowledge about misuse of antibiotics? Do they know that they should not misuse antibiotics?

P2- I don't think they have proper knowledge about misuse of antibiotics. If they had enough knowledge about antibiotics, then they would not have used the antibiotics randomly. I have seen most of the people using antibiotics in these areas.

I- We have seen antibiotic resistance as an emerging problem. Why are we not being able to reduce the antibiotic resistance from the viewpoint of community?
P2- From the viewpoint of community, community people want to be recovered very fast. So, they use and misuse antibiotics.

P1- We also became unable to give a full course of antibiotics [doses of 7 days]. If people take the full course of antibiotics [doses of 7 days] then antibiotic resistance will be reduced. We can see many people who take full dose of antibiotics while we can also find equal number of people who don't take full dose of antibiotics.

I- At the end, I would like to ask you, what is the perception of people regarding antibiotics?

P2- People perceive antibiotic as an effective medicine. They think that if they take antibiotic, then their health problem will be solved very quickly.

P1- People who don't know the meaning of antibiotic, they just don't know that they should take the full dose of antibiotic. Those people who have awareness about antibiotics, they take the full course of antibiotics. There are different perceptions of people about antibiotics according to area, society, education of individual etc.

I- Antibiotic is widely used term but along with antibiotic, there are other antimicrobial drugs and resistance as well such as anti-parasitic, anti-fungal, anti-helminthic drugs and resistance etc. How can we explain these terms to community? How can we ask these terms to them?

P1- The older people of our society use various terms like kufat, bailo [typhoid]. [36:31]   People of =Pyuthan=, =Argakhachi= use these languages. There are some people who don't understand about antibiotics at all. For those people, we have to show them cover of antibiotics to explain them about antibiotics while educated people understand easily about antibiotics.

I- In case of anti-fungal infection, can we use the term "itching problem" to make them understand?

P1- Yes, we can use the term "itching problem" [daadh] to make them understand about anti-fungal infection. For diarrhea, dysentry, madhesi people use the term [prolapse of stomach ] [pet jhareko]. They aslo use the term khajuri for itching problem [scabies].

I- Okay. Thank you so much for your time and cooperation.

P1:Thank you

P2: Thank you

**Interview Transcript 7**

Interview details

| Participant ID | 7 |
| --- | --- |
| Occupation / role | Health post in-charge (health assistant) |
| Gender | M |
| Area | Rural |
| Service provider | Health post |

Index:

I: Interviewer

P: Participant

I- “Namaste! “

P-“Namaste ma’am!”

I- What type of patients mainly visit this health center?

P- All types of patients like patients having fever, pain etc. visit this health center.

I- Is it a clinic?

P- We have clinic as well.

I- Before coming to the clinic, do people already take medicines?

P- If a person comes here from far place, then they already take medicine. But if a person comes here from nearby place, then s/he does not take medicine before visiting doctor.

I- People from how far places come to visit this clinic?

P- People from 1 to 2 km away come to visit this clinic.

I- What kind of medicines are mainly used?

P- Mainly, antibiotics like [cephalexin](https://www.google.com/search?sxsrf=ALiCzsZP0B31lfPv2cA7-MAFGHiLJL4EFg:1654591573007&q=cephalexin&spell=1&sa=X&ved=2ahUKEwiLkNT3-Zr4AhVBAqYKHdwGB4gQBSgBegQIARAy) etc. are used.

I- In what conditions, are antibiotics used?

P- Antibiotics are used in the case of typhoid, when the fever is high, malaria etc. When the condition is normal, antibiotics are not used.

I- Are children also brought in this clinic for treatment?

P- Yes, children are also brought here for treatment.

I- Are the medicines used in children and adults different or same?

P- There are different medicines used for children and adults.

I- How are the antibiotics used in adults and children?

P- The names of antibiotics are same but the doses are different for children and adults.

I- How are the practices of people for using medicines from pharmacy?

P- The practices of people for using medicines from pharmacy are normal. We do not have pharmacy here. We write medicines and send people to pharmacy to buy medicines.

I- People buy medicines in other pharmacies which are not available here.

P- Yes

I- Do people get prescription of medicines from doctor only, or you also give medicines to the patients?

P- We don’t have doctor. I give medicines to the patients. I deal with patients myself.

I- Is it a pharmacy?

P- It is a clinic. We don’t have pharmacy.

I- Is it like a medical?

P- It is a clinic, like a first aid center.

I- Do you keep medicines here?

P- Yes, we keep basic medicines here.

I- Do people come and ask for the name of medicines by themselves?

P- Yes, some people come and ask for the name of medicines by themselves.

I- Do they ask for antibiotics by taking the name of medicine?

P- No, they do not ask for antibiotics by taking their name. But some people ask for antibiotics like =megapaen= etc. I should give them those medicines when they ask.

I- Do educated people ask for antibiotics by taking name of antibiotics?

P- Yes, obviously, educated people are the ones who ask for antibiotics by taking name of antibiotics.

I- Do uneducated people ask for antibiotics by taking different name of antibiotics?

P- No, uneducated people don’t ask for antibiotics by taking different name.

I- Is there any local language for antibiotics for the people in this place?

P- Yes, they ask for medicines of high dose if they want antibiotics. Some people ask for medicines of powder which means antibiotics.

I- Are the medicines of powder which means antibiotics are for children?

P-Yes, the medicines of powder which means antibiotics are for children.

I- That means, people ask for antibiotics directly.

P- Yes, if a person is educated, then s/he directly ask for antibiotics but if a person is not educated, then s/he does not ask for antibiotics. [3:47]

I- When children are angry, mainly who bring them to hospital, is it their mom or dad?

P- Mainly, mother of a child brings him/her to clinic.

I- When you give medicine, do you teach people about the proper use of medicine?

P- Yes, I teach people about the appropriate method to use medicines.

I- If you tell them to take medicines for 5 days, then do they agree?

P- No, they do not take medicines according to the required dose.

I- Why do they not use medicines for required dose?

P- It is because they don’t have enough money. If they take the total dose of antibiotics, then it costs 400 to 500 rupees. But many of them come here only with 50 to 100 rupees. So, they just take medicine for 1 to 2 dose and tell us that they will come tomorrow to take the remaining dose.

I- And, do they come tomorrow, to take the required dose?

P- No, most of the people don’t come to take the required dose. They just take 1 to 2 doses and when the symptoms reduce, they don’t take the medicines.

I- Do you counsel them to eat medicines for all the doses with appropriate methods?

P- Yes, I counsel them to eat medicines for all the doses with appropriate methods.

I- That means, you give antibiotics to people if they come and ask for antibiotics directly.

P- No, I ask them their symptoms. I check their conditions and if necessary, then I give them antibiotics, otherwise, I will not give them antibiotics. [6:18]

I- In which places, are antibiotics found in this community?

P- Antibiotics are found everywhere in the medical shops in =Chauraha=.

I- Antibiotics work for bacterial infections. Except bacterial infections, what are the other problems that people bring in this clinic?

P- Yes, people come with other problems as well like pain, vitamin tablet, syrup etc.

I- Do diarrheal patients also come to this clinic?

P- Yes, Diarrheal patients come to my clinic.

I- What medicines do you give for diarrheal patients?

P- I give antibiotics like ofloxacin for diarrheal cases. [6:41]

I- For anti- fungal medications, what medications are used in conditions like scabies?

P- For anti- fungal medications, I give fluconazole for scabies.

I- Do people buy and take fluconazole themselves?

P- Yes, they ask for ointment. If a person is educated, then s/he may ask for the medicine by name. Otherwise uneducated people also ask for medicines with their colors.

I- When people come to you, you said, some people come already by taking tablets. How do you find out the patients, who have already taken medicines?

P- I ask them whether they have taken medicines or not.

I- Do they bring tablet cover to show the medicines that they had eaten?

P- Yes, they bring tablet and say that they were having that tablet.

I- There are different companies of medicines. There are different generic and brand names of medicines. If we take the packets of medicine to people, do they understand about the medicine?

P- No, they do not understand about the antibiotics. If they are educated, then only they will understand, otherwise, they will not understand. [8:28]

I- We have to ask questions to community people about antibiotics. How can we make them understand about antibiotics? Would it be easier for us if we show packet of medicines to them?

P- Yes, it would be easier to make them understand if you show sample of medicines to them. It would be difficult to make them understand by taking their name.

I- In this community, people of which ethnicity are found more?

P- In this area, mixed group of people live here.

I- For example; are the pattern of taking medicines by hilly people different to the pattern of people of terai about taking medicine?[8:45]

P- Yes, their patterns are different. There is high level of education in hilly people in comparison to terai people. In terai, people directly ask for effective medicine, vaccine etc. People from hill ask for medicine with low dose at first and if it does not work, then only, they will seek for medicines of higher doses.

I- How do community people dispose remaining or left over medicines?

P- Community people throw away left over medicines. They do not keep those medicines at home. They keep the medicines while they are using and when they do not have to use, then they throw away those medicines.

I- Where do they throw those left over medicines?

P- They throw waste medicines in garbage collection site.

I- Do community people keep left over medicines to use those medicines for the next time when they are sick?

P- Some people keep the left over medicines to use those medicines for the next time when they are sick. They come to me and ask if they can eat that medicine or not. They ask me if the medicine has reached expiry date or not. Educated people see the date and use those medicines themselves in case of pain, gastritis etc. because they have some knowledge about the medicine while uneducated come to ask me if they could use the remaining medicine or not.

I- How much population is covered by this clinic?

P- This clinic has covered 10 to 15 villages and provide them with clinical facilities.

I- Do people from urban areas come to this clinic to get its services?

P- No, urban people get various better health facilities in urban area so they do not come here.

I- We have already heard about antibiotic resistance. What do you think are the reasons behind antibiotic resistance?

P- The reasons behind antibiotic resistance are discontinuous use of antibiotics. People do not take the required dose of antibiotics and their intake of antibiotics is also irregular and discontinuous which is not according to the requirement. So, antibiotic resistance occurs in people.

I- Okay, thank you for your time and cooperation.

P- Okay. thank you.

**Interview Transcript 8**

Notes taken after the interview, transcript not available

**Interview Transcript 9**

Interview details

| Participant ID | 9 |
| --- | --- |
| Occupation / role | Community resident |
| Gender | F |
| Area | Urban |
| Service provider | N/A |

Index:

I: Interviewer

P: Participant

I- “Namaste”- From the viewpoint of community, community people want to be recovered very fast. So, they use and misuse antibiotics.

P-“Namaste”

I- When you are sick, where do you go to examine?

P- I go to government hospital when I become sick.

I- Do you go to district hospital?

P- Yes, we have insurance and government hospital only gives the free treatment facilities for insured person.

I- when you have simple fever, common cold, then in which health care center, do you go?

P- We go to the hospital during simple fever, common cold as well.

I- Are there medical shops nearby?

P- Yes, here is medical shop nearby. Sometimes, we go to medical shops to buy simple medicines during common cold, etc. In case of other problems, we go to government hospital.

I- When you go to medical, how do you request for medicines?

P- I take insurance card when I go to government hospital but when I go to medical shops, I ask him/her to give medicines when I have headache, cough, fever etc.

I- Then, do they give medicines themselves?

P- Yes, they give medicines like paracetamol etc. themselves.

I- Does he explain you about the medicines and the methods to use the medicines?

P- Yes, he explains me about the medicines and the methods to use the medicines. He also belongs to government hospital.

I- As you said earlier that, you go to medical shops during simple health problems like fever, cough etc. and during complex health problems, you go to government hospital. What do you mean by complex health problems? In which health conditions do you go to government hospital?

P- Complex health problems which I may suffer are high fever, body pain, severe cough and cold etc. In these conditions, I go to government hospital.

I- When you go to hospital, does doctor prescribe medicines?

P- At first, doctor examines me and give medicines according to necessary conditions.

I- When s/he prescribes medicines, does s/he explain about the mentioned medicines, their required doses, methods to take the medicines?

P- Yes, s/he explains me about the mentioned medicines, their required doses, methods to take the medicines. S/he gives me medicines for 4,5 days and tells me that we will observe the result of medicine and if it doesn’t work, then we will add or change the medicines. [3:47]

I- How many members are there in your home?

P- We have 8 members in our home. We have 4 small children under 5 years of age. One grandson is about 5 to 6 years old. One child has just been born. One child is under 3 years of age. One granddaughter is 11 years old.

I- When children are sick, then where do you go for checkup and treatment?

P- When children are sick, my son takes them to hospital in =Butwal=.

I- Have you done insurance of children?

P- Insurance have been done for all grandsons and granddaughters. Children are taken to hospital in =Butwal= after referring from the hospital nearby.

I- Then the first contact point is hospital nearby.

P- Yes

I- Do you take children to medical shops nearby for simple health problems?

P- No, we don’t take children to nearby medical shops because children should be given proper treatment which is not available at local medical stores.

I- You have a son. You have a daughter in law as well… [interrupted]

P- One daughter in law is at abroad. One daughter in law has just given birth. So, she has went to her mother’s home to take rest.

I- When family members get sick, do different family members go to different hospitals?

P- No, we go to same government hospitals.

I- When children are sick, who take them to hospital?

P- When children are sick, my elder son takes them to hospital.

I- Where is the mother of children?

P- She is in abroad.

I- In which country has she gone?

P- She has gone to Saudi Arabia.

I- Do you follow the advice of doctor and give medicines according to the required doses and directions of doctor?

P- Yes, I follow his advice of doctor and fulfill the required doses.

I- For example; if the doctor tells to take the medicines for 7 days, then do you fulfil the required dose?

P- Yes, I buy the medicines for 7 days according to the required dose and take the medicines according to the directions of the doctor.

I- Sometimes, medicines are left over or remained after the use. For example: medicines like cetamol, medicines of common cold etc. What do you do with the remaining useless medicine?

P- I throw away those remaining medicines.

I- Do you re-use those medicines?

P- No, we don’t reuse the medicines. Those medicines are damaged and have negative impacts on health. I throw those medicines. I do not keep those medicines for further use.

I- Where do you dispose or throw the waste medicines?

P- I throw the waste medicines anywhere around the home.

I- Are there animals in your home?

P- No, I don’t have any domestic animals at home.

I- Whose chickens are these?

P- These are my chickens. There are 4 chickens. That’s all I have. I don’t have other animals.

I- You have made a big home for children?

P- Yes. My sons work and these [house, land] are the contributions of my husband.

I- That means, you don’t have animals like goat at home.

P- No, I don’t have goats. I have been old. I cannot work more. Sons don’t stay at home.

I- When you feel sick, do you take advice from family members, relatives?

P- My family members take me to hospitals. They don’t give me much advice.

I- Do people give you any advice like using some medicines which have been beneficial for them during any health problem?

P- No, they don’t give me such suggestions. They take me to hospital.

I- As you said earlier that you have heard about cetamol. Have you heard name of any other medicines beside cetamol?

P-No, I have not heard about names of medicines.

[ Another participant arrives who is the relative of first participant.]

I- Hello sister, I would like to ask you a question which was little bit difficult for mother.

P- Okay

I- When we and our children are sick, we use many medicines. Do you know name of any other medicines besides cetamol?

P- I don’t have much information about name of medicines.

I- Have you heard about antibiotics?

P- Yes, I have heard about antibiotics. Antibiotics should be used when we are sick.

I- What do you think are the differences between cetamol and antibiotics?

P- I think when cetamol does not reduces the health problem, then antibiotics are used.

I- How do you use antibiotics when you are sick? [12: 12]

P- When we get sick, we go to medical shop and do checkup. Then the medical personnel give us antibiotics.

I- Is doctor available in medical shop?

P- For small children, my brother brings medicines from =Butwal= hospital by consulting with pediatric.

I- That means, you always consult doctor before using antibiotics for children.

P- Yes

I- How do you use antibiotics for adults?

P- For adults, we go to medical shop and bring medicine. If the medicine from medical shop don’t work, then only, we go to hospital for further treatment.

I- In case of throat pain, what types of medicines do they give?

P- They give cetamol, pain killer, nims for pain.

I- When you go to medical shops saying you have throat pain. Do they give you counselling while giving medicine about the medicine, their types, methods of using the medicine?

P- Yes, they give us information about the medicine, methods of using the medicine. For example: medicine for throat pain is taken once in a day either in the morning or in the afternoon. There are 3 tablets for throat pain. If the medicine does not work in 3 days, then another medicine is given. If the medicine doesn’t even work in second attempt, then s/he suggest me to go to hospital and do the checkup by consulting with doctor. They tell me that if tonsil is not treated for long time, then it may cause infection in the throat. It may create a lumpy mass inside the throat which may take a long time to heal.

I- Does the medical practitioner tell you that they have given antibiotics to you?

P- Yes, they say that they have given antibiotics to me.

I- You have understood about antibiotics. Do people around in this place also understand about antibiotics?

P- Yes, community people understand about antibiotics.

I- As you said, antibiotics are used in tonsil, fever. Likewise, have you heard and used antimicrobial drugs for itching problem, ascariasis etc.?

P- I eat medicine for ascariasis in every 6 months because when we suffer from ascariasis, we feel pain in the stomach around the navel and have nausea and vomiting.

I- Have you used medicines for rashes and itching problems?

P- No, I have not used the medicines of rashes. But I have problem of throat allergy. So, I use tablet for throat allergy. I had also examined my nose in hospital of Kathmandu. Doctor had told me to come to hospital in every 6 months. I used to go to hospital often but after sometime, I did not go to hospital. Doctor had said me that there was no need of surgery in my case but I should take medicines regularly and he had given me various types of mediicnes.

I- Mother had said that there are 4 chickens. Who take care of these chickens?

P- Mother takes care of our 4 chickens. If mother goes somewhere, then we will see the chickens.

I- When chickens are sick, how do you treat them?

P- We take chickens to veterinary [ Animal Service Office] when they are sick. Animal Service Office is nearby so it is easier for us to get the medicines.

I- Do they explain you about the type of medicines, their direction of use?

P- They give us medicine of ascariasis. They give us capsules, vitamins, powder medicines.

I- From where do you bring the grains for chickens?

P- We give them grains [wheat, rice] of our own home because it is very expensive to buy grains for chickens. These chickens eat grass a lot along with grains. [17:56]

I- As you said earlier that you buy medicines and eat but sometimes, medicines are left after using. What do you do with the left over medicines?

P- We throw the waste medicines otherwise children may eat.

I- Where do you throw waste materials?

P- Waste medicines and materials are disposed using fire.

I- Do you dispose organic waste materials in compost and others using fire?

P- Yes, we collect waste materials in free land and set fire on the waste materials so that the land would not look dirty.

I- Do you follow the prescription of doctors and pharmacist and buy full dose of medicines?

P- Yes, I follow the prescription of doctors and pharmacist and buy full dose of medicines. If my doctor tells me to take medicines for 7 days, then I will take medicines for 7 days.

I- If the medicine does not work effectively even after taking 7 days, then what will you do?

P- I will go to the doctor and tell him/her that the medicines have not shown positive effects.

I- Sometimes, when we use medicines for 2,3 days, it feels like our health problems have been solved. In such case, do you continue the medicines up to 7 days or leave it in 2,3 days?

P- I continue the medicines up to the required dose. I don’t leave the medicines randomly without completing the dose because if I leave the medicines without completing the dose, then the problem may reappear again. Doctor won't give medicine randomly. S/he will give the complete dose which should be taken by the patients.

I- Mother had said that when children become sick, son take them to hospital. Who takes care of the children most of the time? [19:53]

P- We, all the members of family take care of the children. We have a complete family.

I- If health problems are seen in chickens, then who will take them to veterinary hospital?

P- Mother will take chickens to veterinary hospital if any health problems are seen.

I- Who give medicines to chickens?

P- We [Mother and me] give medicines to chickens if they get sick. Medicines should not be given individually to chickens. If medicines are mixed with grains, then they will eat easily.

I- But one must remember the dose required to give chickens in appropriate quantity.

P- I tell the appropriate quantity to mother and she gives the medicine to the chickens.

I- In this area, which animals are usually domesticated in home?

P- Buffaloes, goats, hens are usually domesticated in home in this area.

I- Are these animals common in this area?

P- Yes, these areas are very common in this area. There are villages nearby where madhesi people live. Buffaloes are kept in every home in madhesi people nearby.

I- Some quantity of medicines of animals may also be left after their use. What do you do with the left over medicines of animals?

P- We check the date of left over medicines and keep it for the re-use otherwise we throw the medicines if the date of the medicines is expired?

I- When do you use the remaining medicines with dates?

P- We use the remaining medicines with dates during the next time when animals get sick. Before giving medicine to animals, we check the date and consult the veterinary doctor. If the doctor says that the medicine can be used then, the medicines will be used otherwise, we throw the medicines.

I- How do you manage waste materials of chickens?

P- We use waste materials of chickens as compost manure in field to grow crops.

I- Do we have drainage system for waste materials of toilet?

P- We use septic tank for the collection of wastes of toilet.

I- How do you dispose waste water used for washing clothes, dishes?

P- We throw away waste water from washing clothes, dishes etc. in kitchen garden.

I- We just wanted to know the use of antibiotics.

P- Children have tonsils frequently. Small baby frequently have throat problem. If they are taken to hospital, their half problems will be solved by meeting doctor.

I- How do people understand antibiotics?

P- People think like antibiotics will solve their health problems.

I- Who takes care of buffaloes most of the time in this area?

P- Every member of their [Madhesi people] family takes care of buffaloes because they sell milk which is their main income source.

I- Do you think you have used antibiotics in chicken knowingly or unknowingly?

P- Our mother give herbs to chickens if they get sick. If they do not recover using herbs then she takes them to veterinary hospital.

I- That’s all I had to ask. Thank you so much for your time and cooperation.

P- Thank you.

**Interview Transcript 10**

Interview details

| Participant ID | 10 |
| --- | --- |
| Occupation / role | Community resident |
| Gender | F |
| Area | Rural |
| Service provider | N/A |

Index:

I: Interviewer

P: Participant

I- “Namaste”

P- “Namaste”

I- What do you do when members of your family fall sick with simple health problems like fever, common cold?

P- During simple health problems, we stay at home, take home remedies like hot water, jwano, turmeric water, fresh food, vegetables etc. We avoid eating stale food or unhealthy food.

I- If health condition is not getting better, then what is your first contact point to health services?

P- I go to district hospital at first in such condition.

I- Do you take medicines according to the prescription of doctor?

P- Yes, I take medicines according to the prescription of doctor.

I- Do you understand and have knowledge about medicines prescribed by doctor?

P-No, I can’t understand about medicines prescribed by doctor.

I- That means, you show the prescription paper to pharmacist and bring medicines which are given by pharmacists.

P- Yes. I also take medicines to doctor and make sure that the medicine is correct as prescribed by the doctor.

I- Do you know the type of medicines which you are using like antibiotic, anti-viral medicine, anti-helminthic medicine etc.? For example, the medicine used in daadh [itching problem], scabies etc. Do you know about the categories of drugs?

P- I ask doctors about the type of medicines. Many farmers in the village don’t understand about the medicines given by doctor. For this, people must be experienced, have knowledge about various problems.

I- Do you remember, when did you take antibiotics for the last time?

P- I used antibiotics in Bhadra, last year. COVID-19 was at peak at that time. I was suffering from fever.

I- Yes, at that time, COVID-19 was at peak.

P- Yes, I was suffering from cold at that time. I had symptoms of sneezing, runny nose, cough. I have cold allergies and allergies due to dust.

I- Do you remember the medicine given by doctor at that time?

P- Doctor gave me antibiotic at that time.

I- How long did you need to take antibiotics at that time? Do you remember the name of antibiotic?

P- The course of antibiotics was 7 days. No, I don’t remember the name of antibiotic resistance.

I- Did you take the full course of antibiotics?

P- Yes, I took the full course of antibiotics.

I- Sometimes, people either forget to take antibiotics or after sometimes, when people get recovered, then they stop taking antibiotics.

P- I don’t leave medicines. I take medicines to full doses. I teach other people as well about the importance of taking full course of antibiotics.

I- Did your health condition get better after taking that medicine?

P- Yes, my health condition improved after taking the medicine. [6:35]

I- Have you reared domestic animals at home?

P- No, I have not reared any domestic animals at home.

I- In this area, do people rear domestic animals?

P- Yes, people in this area rear domestic animals in every house.

I- You have worked in women and children organization, right?

P- Yes, I had worked in women and children organization.

I- That means, you have gone to this village, tole, area.

P- Yes, in Kapilbastu, there is no place where I have not gone.

I- Do you know what medicines are given to animals when they get sick?

P- I had taken training of 1 month related to animal given by veterinary doctor when I was working in the women and children organization. [7:33] I used to make reports and submit those reports to the doctors.

I- Do farmers know about medicines which should be given to animals for any specific health problems of animals?

P- No, most of the farmers do not know about medicines for specific health problems of animals.

I- What do farmers do with the left over medicines of animals?

P- They keep the medicines for further use, to give the animals if they get sick after sometimes.

I- Do you know what kind of medicines are given to plants?

P- There are various types of grass given to animals.

I- Do you put insecticides and pesticides to plants?

P- No, I do not use any insecticides and pesticides to plants.

I- How much insecticides and pesticides are used by community people in plants?

P- Yes, a lot of insecticides and pesticides are used by community people in plants. Women should be reached in decision making position to solve all the problems.

I- How much women’s participation do you see in the agricultural practices, taking medicines from shop etc.?

P- No, there are not much women’s participation in agricultural practices, taking medicines from shop etc. I see male domination in every sectors of the society. Women are always dominated by men. Comparing to past, women have come forward. We have also made various organizations for women.

I- Where do people dispose waste materials of animals, dead bodies of animals in this area?

P- People used to throw the waste materials of animals, dead bodies of animals randomly in this area. We have told people about the negative consequences of the unmanaged disposal of waste materials, animals in community. When we spread the awareness, then people became little aware and all of the community people disposed waste materials in a united way. But people are still unmanaged and they are not being able to manage the waste materials and dead bodies of wild animals. Various conflicts arise in the community in the disposal of waste bodies, waste materials etc.

I- Do people also throw the expired medicines in the dumping of waste materials?

P- Yes, they put everything in the dumping of waste materials including expired medicines, sanitary pads and all other wastes.

I- What may be the cause of lack of management of waste materials in this place?

P- People in this area are very ignorant and unaware. At present condition, the situation is improving. At past, there was open defecation practices in this area. Roads used to be covered with human excreta. Municipality forced them to build toilet and end open defecation practices in every house. People used to say that they can’t defecate in toilet. There were wide spread superstitious beliefs as well like daughter in law should not use the toilet used by father in law etc. To make this place free from open defecation, municipality, police have worked a lot and now, the open defecation practices have ended. In the rainy season, road has been made. Drainage system has not been made properly. There occurs flooding in rainy season.

I- What kind of measures should we adopt to reduce the use of insecticides and pesticides in crops?

P- People should be made aware about the negative effects of insecticides and pesticides on health. Natural manure like cow dung should be used in crops instead of insecticides and pesticides in the field to grow crops.

I- What can be done to increase community participation to reduce the use of insecticides, pesticides, chemical medicines?

P- To reduce the use of insecticides, pesticides, chemical medicines, community people should be made aware. Educated people should share their knowledge to their family and make them aware about the waste management, misuse of pesticides, chemical medicines etc.

I- To do awareness raising programs in this area, should we go to community people by saying that, “we are from Kathmandu and we are here to conduct awareness raising programs” or should we mobilize the local human resources to increase awareness level of people?

P- Local human resources must be mobilized to conduct any programs effectively because you guys who come from various organizations from Kathmandu many not know about the local language, culture, values and beliefs of community people in this locality, so community people should be mobilized.

I- How long does it take to aware people about antibiotics, antimicrobial resistance, harmful effects of pesticides and insecticides?

P- It takes a long period of time to aware people about antibiotics, antimicrobial resistance, harmful effects of pesticides and insecticides. Just to end the open defecation practices, municipality, police had to work so hard to change the behaviors of people. So, to change the behavior of people, it takes a long period of time.

People in this area have low socio-economic status as well. People are involved in agriculture, animal husbandry etc. They earn little money by working very hard in field and by rearing animals. So, they don’t to spend their money in a random manner as well. Domestic animals eat all the green crops and it may also lead to scarcity of crops for human beings in this area. Government is also not aware about the problems of people.

I- Do people in this place go to hospital regularly during any health problems?

P- Educated people go to hospital during any health problem but uneducated people also don’t have economic status to go for regular medical checkup and treatment.

I- Can people in this area afford medical treatment or not?

P- People with high economic status go for treatment. People with low economic status only go to hospital at the end stage because they cannot afford medical treatment. People are not much aware about the balanced diet, food and nutrition practices which are required for the healthy life.

There are lack of management in every development sectors like transportation, drainage system, waste management etc.

I- One type of problem is lack of economic status of people due to which they cannot afford proper medical treatment. Another type of problem which is seen is anti-microbial resistance which means people are using anti-microbial medicines like antibiotics, anti-fungal, anti-helminthic, anti-protozoal medicines, pesticides, insecticides, chemical medicines in any health problems of human beings, animals as well as in farming and agriculture. People are using so much antibiotics to them and their animals, in any health problems that anti-biotic resistance is increasing day by day. And now, anti-biotic medicines are also not working to solve the bacterial infections in human beings and animals. What is the condition of anti-microbial resistance in this place?

P- Doctors give antibiotics to sick people to treat them and make them free from infections quickly. People also want to get quick relief from health problems so they also want to use antibiotics in every health problem. But doctors should tell their patients about the positive and negative effects of the medicine sand antibiotics that s/he is giving to the patients. Proper follow up should be done to patient to check his/her health condition and to complete the course of treatment. Doctor should tell the patient to complete the course of treatment and take the full dose of medicines as prescribed by the doctor.

I- Are there people who take antibiotics from medical shops without the consultation with doctor?

P- Yes, there are a lot of people who go directly to pharmacist and ask for different medicines and antibiotics without visiting doctor. They may also have low economic status to afford medical treatment and doctor’s fee. People think that it will be cheaper and less time consuming to go to medical stores directly than to go to visit doctor in hospital. Medical practitioners also take the people as their business clients and give them antibiotics and medicines according to their will for 1,2 days without taking care of the full doses and course of antibiotics. People also don’t have awareness that they should regularly visit doctors before taking antibiotics and other medicines.

I- It’s okay mother. Thank you so much for your time and cooperation.

P- Thank you.

**Interview Transcript 11**

Interview details

| Participant ID | 11 |
| --- | --- |
| Occupation / role | Community resident |
| Gender | M |
| Area | Rural |
| Service provider | N/A |

Index:

I: Interviewer

P: Participant

I- “Namaste”

P- “Namaste”

I- In case of simple health problems like fever, common cold etc., how do you solve your health problems?

P- We do treatment in case of fever, common cold and other health problems.

I- Which health center do you go during such health problems?

P- I go to near health centers for the treatment. If treatment does not occur, then I go to the hospital of =Taulihawa=. Pokhara

I- Do you go to health centers during simple health problems like common cold?

P- I don’t go to health centers during simple health problems like common cold. I go to medical store in such condition and take medicine from there.

I- Do you consult with doctor before taking medicines?

P- Yes, I consult with doctor and take medicines according to his/her advice.

I- Do you take antibiotics when you get sick?

P- Yes, I take antibiotics when necessary.

I- Do you know name of some antibiotics?

P- No, I don’t know the names of antibiotics. Doctor prescribes me antibiotics and I take it but I don’t know their names.

I- How much antibiotics do you take in a year?

P- I don’t usually take antibiotics. In severe cases, I take antibiotics. My children take antibiotics when they get sick. [3:6]

I- These children look small. Are these children yours?

P- Yes, these are my children.

I- How old is she?

P- She is 3 years old.

I- Is she a girl?

P- Yes, she is a girl.

I- Children usually get sick under 5 years of age.

P- Yes, that is true.

I- What are the common health problems faced by your children?

P- They suffer from fever, stomach ache, diarrhea, cholera etc.

I- Do you or your wife know more about health problems faced by children?

P- Yes, my wife knows more about health problems faced by children. She is the one who gives medicines to children when they get sick.

I- Who goes to health centers to take medicines for children? Is that you or your wife?

P- Health centers are nearby. So, my wife also goes to take medicines.

I- If the children don’t get recovered here. Then, where do you take the children?

P- If the children don’t get recovered here, then I take my children to hospital of =Taulihawa=. I take my sick children to =Jahabed’s= health center if they don’t get recovered here. And from there, I take my children to hospital according to the advice of =Jahabed=.

I- We are carrying out health investigation program from an organization. This organization is working for the social, health issues etc. So, we are trying to find out the activities and health behaviors of people to bring suitable programs in community level by involving community people to improve the health condition of people. We are asking you about antibiotics because this project is mainly concerned about use and misuse of antibiotics by community people. Antibiotics are widely used not only in this place, but also in all over the world. Due to the overuse and misuse of antibiotics, antibiotics can be ineffective in future. So, this program is launched to find out the anti- microbial resistance in community people. =Kapilbastu= district has been selected to do the investigation about anti- microbial resistance. This research is also being conducted in Bangladesh. The government of UK [United Kingdom] conducts health investigation all over the world. Under this, HERD International has some responsibilities to conduct health research. We are the officers of HERD International. So, we came here to conduct the study. You may have curiosity about these issues. So, I am giving you information about this program. Health research teams will come to community time and again to conduct the study.

Now, let’s talk about anti-microbial use and resistance in animals. Do people use antibiotics in domestic animals like cows, chickens, goat etc.?

P- Yes, people use antibiotics when domestic animals like cows, chickens, goat get sick. They may suffer from fever etc. People go to agro vet store to bring medicines for animals.

I- Have you reared domestic animals?

P- I have only reared goats in my house.

I- Do you take medicines from agro vet store when your goats fall sick?

P- Yes, I take medicines from agro vet store when your goats fall sick

I- Don’t you consult veterinary doctor before giving medicine to goats?

P- At first, I go to agro vet store and take medicine and give it to goats. Most of the time, my goats get fine after giving medicine from nearby agro vet store.

I- Does s/he investigate what has happened to goats before giving medicines in agro vet store?

P- Yes. s/he asks me about the health problems that my goats are facing. If the problem is complex, then s/he comes to my home to check the goats and give medicines after checking.

I- Do you know name of those medicines given by medical person?

P- No, I don’t know the names of medicines given by medical person.

I- Are you involved in farming?

P- I do farming of rice, wheat etc. in a small quantity.

I- Pests may attack crops. Do you use pesticides in your crops?

P- I don’t put insecticides and pesticides in rice, wheat etc. but in vegetables, if pests are seen, then I use pesticides.

I- Do you know the name of those pesticides?

P- No, I don’t know the names of pesticides. I use pesticides given by medical person in agro vet store.

I- How do you dispose the remaining pesticides?

P- I throw the remaining pesticides and insecticides in river or bury the medicines and pesticides in soil.

I- Do you bury the pesticides in soil as well?

P- Yes, I bury them in soil.

I- Does municipality send vehicle here to collect the waste materials?

P- Vehicle of municipality does not come here to collect the waste materials.

I- Then, how do you dispose the waste materials of your house?

P- People dispose their waste materials in their home by themselves by burning them or burying them with soil.

I- How do they dispose animal wastes like cow dung etc.?

P- People use animal wastes as manure for agriculture.

I- That means, if we conduct any discussion program in your community, then do people understand about antibiotics?

P- No, they don’t understand about antibiotics.

I- If we show them pictures of antibiotics or picture? [interrupted]

P- Yes, people can understand medicines if picture or bottle of medicines are shown.

I- In your opinion, when should we use antibiotics?

P- We should take antibiotics when we get severely sick.

I- Where do you go to take medicines which are not available here?

P- If I don’t get medicines here, then I go to =Taulihawa= to take the medicine. If I don’t even get medicine in =Taulihawa=, then I go to border side to take the medicine.

I- Most of the time, who goes to take medicine from family?

P- The guardian of house, father goes to take medicines.

I- If children should be taken to hospital, then who take them to hospital?

P- I take my children to hospital myself.

I- How many children do you have?

P- I have 5 children. I have 4 daughters and 1 son.

I- That means, you need medicines frequently when the children are sick?

P- Yes

I- These are the issues that I had to ask you. Thank you for your time.

P- Thank you.

**Interview Transcript 12**

Interview details

| Participant ID | 12 |
| --- | --- |
| Occupation / role | Female community health volunteer (FCHV) |
| Gender | F |
| Area | Urban |
| Service provider | N/A |

Index:

I: Interviewer

P: Participant

I- “Namaste”

P- “Namaste”

I- I- What kind of health problems are mainly seen in people of this community?

P- Some women may have problem of uterus; some may have problem of having early menstruation. Some woman may have swollen legs during pregnancy. Some women may have problems while having iron tablets. They have many problems in them but they don't share us their problems directly. Some may have bleeding during early pregnancy. Some women may have dehydration in pregnancy.

I- These are main problems of women. Besides the women health problems, what are the other health problems that people in our community face?

P- There are various health problems like fever, typhoid, common cold, itching problems etc. prevalent in our community.

I- What is the first contact point of community people during health problems?

P- If people meet us, then they share their health problems with us otherwise they visit health centers. [1:51] If people have complex health problems, then they visit hospitals in =Butwal=.

I- Do people buy medicines themselves?

P- Yes, people buy medicines themselves. If people go to health posts, then medicines are given to them freely otherwise, they have to pay for medicines if they buy medicines outside the health post.

I- Do people go to pharmacy and medical shops to get medicines without visiting doctor?

P- Yes, a lot of people go to pharmacy and medical shops to get medicines without visiting doctor.

I- Do you have any idea about medicines that people buy from pharmacy or medical stores?

P- People take medicines according to their health problems like fever, common cold, stomach ache, vomiting, diarrheal diseases etc.

I- How much antibiotics are used by community people, by buying from pharmacy, without visiting doctor?

P- Yes, people buy and use a lot of antibiotics. When people go to medical stores, most of the time, they are provided with antibiotics. [2:45]

I- What are the differences between priority of health centers in adults and children when they get sick?

P- Priorities and choices depend on individuals. Some people may bring adult or children to government health center. Some people take children to Taulihawa hospital. Some people and children are taken to medical shops during health problems.

I- In most of the cases, where are children taken during health problems?

P- Children are mainly taken to health center at first. A lot of adults go to medical store during health problems.

I- Can FCHVs [Female Community Health Volunteers] distribute antibiotics according to rule?

P- No, we do not have responsibility to distribute antibiotics. We only distribute cetamol, zinc, iron tablets for pregnant female, ORS [Oral Rehydration Solution] etc.

I- At a time, there was high prevalence of pneumonia and I think, FCHVs [Female Community Health Volunteers] were given responsibilities to distribute antibiotics.

P- In past, FCHVs [Female Community Health Volunteers] were given an antibiotics called cotrimoxazole to distribute in community but now, that program has been finished. Now, we only have simple medicine like cetamol etc.

I- Do people in this community understand about antibiotics?

P- Some people who are educated understand about antibiotics while some people who are not educated, they don't understand.

I- In this area, I think, all people are educated. Aren't they?

P- Yes, people in this area are all educated.

I- In your working area, are people educated?

P- No, in my area, adults and old people are not much educated. But now, new generation are educated. That society is mixed with educated and uneducated people.

I- In that area, if we have to explain about antibiotics, then how can we explain people about antibiotics?

P- We can just tell them about antibiotics. They will understand about its meaning. If we explain about antibiotics to people, they will understand.

I- Even when we take cover of antibiotics, it may be difficult because cover of antibiotics may differ according to brand and company. So, it's being difficult for us to explain people about antibiotics and know their perceptions about antibiotics.

P- You have to go to community people and communicate with them in order to make them understand about antibiotics. [6:20]

I- Is there any local name used for antibiotics or cetamol?

P- No, there are no specific local names for antibiotics or cetamol.

I- Antibiotics are used in bacterial infections. Do people understand about other medicines like anti-fungal medicines used in itching problems like scabies [daadh], anti-parasitic, anti-helminthic medicines for diarrheal disease?

P- Yes, people  understand about other medicines like anti-fungal medicines used in itching problems like scabies [daadh], anti-parasitic, anti-helminthic medicines for diarrheal diseases.

I- What are the places here, where people can go and buy antibiotics and have antibiotics very easily?

P- People go to medical store, which are run by AHW [Auxiliary Health workers] etc. and consult with them to use antibiotics.

I- In which conditions, are antibiotics widely used?

P- No, I don't have much idea about that.

I- Maybe, it is not much your field of interest?

P- Yes, Our main field of interest is Maternal and Child Health [MCH].

I- That means, you don't have much idea about the widely used antibiotics.

P- Yes, I don't have much idea about the widely used antibiotics.

I- I wanted to know about the use of antibiotics in this community.

P- People widely use antibiotics in this area because when people have problems, they go to medical stores where they are always provided with antibiotics.

I- Antibiotics are given and health workers also give information to people about required doses and directions of use. Do people follow the advice and use antibiotics according to the required doses and directions?

P- I don't have much idea about people taking medicines given by health workers but we give them suggestions and tell them to use medicines according to the direction and advice of doctor, according to the required doses. When I ask them, they always tell that they take medicines to full dose according to the prescription of doctor but we may not know the reality.

I- Which regional groups are found more in this community?

P- Hilly people are found more in this community. There are few terai people, otherwise, most of the people are from hill.

I- Are there any differences about priorities of health services and health centers during any health problem between terai and hilly people?

P- No, here are not much differences between priorities of health services and health centers during any health problem between terai and hilly people. In this area, all people are educated.

I- In place where you work?

P- That place consists of terai people. They go to medical store during any health problem, at first. In this place, here are hilly people. They go to district hospital or in =Butwal= hospital.

I- What are the reasons behind these differences?

P- The reason for these differences in priorities of health services is due to education. Here, people are educated so, they don't go to medical store directly during health problem. They go to hospital at first. In village area, they don't have much knowledge so they go to hospital only at the last stage.

I- Do people buy full dose of medicines for like 7 days as prescribed by doctor?

P- I don't have much idea about this condition.

I- How do you dispose or utilize the left over medicine?

P- If the medicines are used according to the advice of doctor, then medicines are not left. People in this area use medicines according to the advice of doctor. so, medicines are not left after using them to full dose.
I- Have you heard about misuse of antibiotics and its negative consequences?

P- No, I have not heard about misuse of antibiotics and its negative consequences.

I- It’s okay. Thank you for your time and cooperation.

P- Thank you.
